# Drought Stress Mediated Changes in Food Crops: Mechanisms and Remediation Strategies

**DOI:** 10.1002/advs.77194

**Published:** 2026-09-16

**Authors:** Xiaoyi Duan, Litao Wang, Tuuli‐Marjaana Koski, Lizhe An, Thomas Efferth, Yujie Fu

**Affiliations:** ^1^ State Key Laboratory of Efficient Production of Forest Resources Beijing Forestry University Beijing China; ^2^ The College of Forestry Beijing Forestry University Beijing China; ^3^ Ecological Observation and Research Station of Heilongjiang Sanjiang Plain Wetlands National Forestry and Grassland Administration Shuangyashan China; ^4^ School of Biological Sciences and Technology of Beijing Forestry University Beijing China; ^5^ Department of Pharmaceutical Biology Institute of Pharmaceutical and Biomedical Sciences Johannes Gutenberg University Mainz Germany

**Keywords:** climate‐resilient agriculture, drought stress, flavonoids, nanomaterials, rhizosphere microbiome

## Abstract

Climate change has intensified drought frequency and severity, threatening global food security, particularly for staple crops like maize, wheat, and soybean. As central secondary metabolites, flavonoids orchestrate cellular redox homeostasis, stress signal transduction, and metabolic plasticity during plant drought responses. Previous reviews are confined to isolated molecular cascades and separate treatment of flavonoid metabolism, rhizosphere microecology, and agronomic mitigation strategies, failing to establish an integrated multi‐scale regulatory framework. To fill this fragmented gap, this review integrates advances in plant physiology, microbial ecology, and nanobiotechnology to construct a cross‐scale framework of flavonoid‐mediated drought resistance in major food crops. We systematically summarize species‐specific flavonoid regulatory networks and metabolic reprogramming triggered by drought‐induced oxidative stress, dissect rhizosphere microbiome effects on flavonoid biosynthesis and drought signaling, and elaborate novel mechanisms whereby nanomaterials reshape flavonoid metabolism and boost drought tolerance via tuning ROS homeostasis. Furthermore, this work integrates soil amendment and precision irrigation to decipher synergistic drought‐resistance crosstalk among agronomic practices, crop metabolism, and root‐associated microbiota. Collectively, this review unifies molecular, microbial, technological, and agronomic perspectives to establish a multi‐scale, interdisciplinary framework for crop drought adaptation. It delivers fundamental theoretical support for climate‐resilient agriculture and outlines priority research avenues to safeguard global food security.

## Graphical abstract

This work presents a multi‐scale framework for flavonoid‐mediated drought tolerance in staple food crops, integrating ROS redox regulation, antioxidant defense, rhizosphere microbial interactions, and sustainable agronomic practices. This interdisciplinary model provides core insights to develop climate‐resilient cropping systems and enhance global food security.

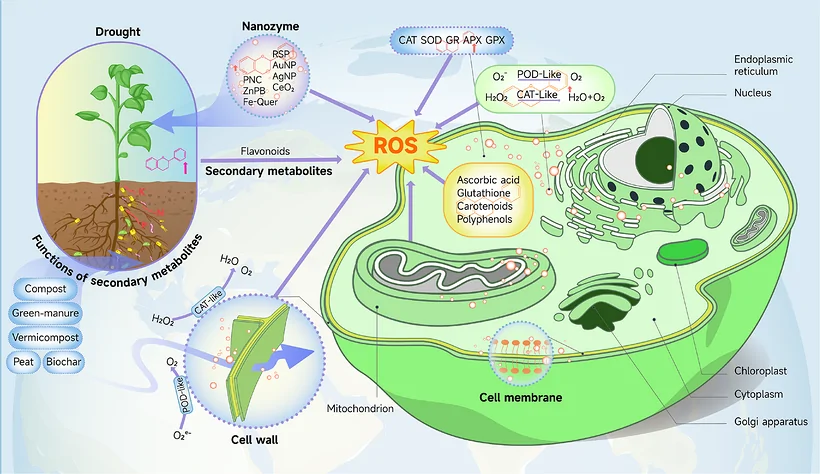

## 1 Introduction

Climate change affects global water supplies by altering the overall water circulation, thereby altering the global water balance and leading to more frequent or extreme weather events [1]. It is estimated that for every 1°C increase in surface temperature, the water vapor content in the lower troposphere increases by about 7% [2], which will enhance atmospheric water holding capacity and exacerbate hydrological extremes, resulting in greater abiotic stress on global crop production. Water availability directly determines crop phenological processes and agricultural production levels. More than 40% of global corn (*Zea mays* L) and 60% of wheat (*Triticum aestivum* L) are grown in areas highly dependent on natural precipitation [3]. In such regions, limited or erratic rainfall during critical growth stages frequently induces severe soil water deficits, with detrimental consequences for food production: aggravated poverty, malnutrition, population displacement, and widening social inequality [4]. The socioeconomic toll of drought is immense: in the United States alone, drought caused approximately $250 billion in economic losses and nearly 3000 deaths between 1980 and 2020, ranking it the costliest and deadliest natural hazard over that period [5]. At the global scale, the impact is even more staggering—between 2019 and 2020, drought severely affected at least 1.43 billion people worldwide, with total losses amounting to $764 billion [6].

Climate change triggers complex environmental perturbations, and plants perform core functions in sustaining ecosystem balance and buffering adverse climatic impacts [7]. Elevated CO_2_ exerts dual context‐dependent regulatory effects on crop water and carbon metabolism. Under mild drought, CO_2_ fertilization drastically boosts photosynthetic carbon sequestration and leaf area index (LAI). Although stomatal conductance at the single‐leaf scale declines, expanded total transpiring leaf area offsets leaf‐level water conservation, leading to increased canopy evapotranspiration. Concurrently, whole‐plant water‐use efficiency and carbon fixation capacity are enhanced, forming a buffering system to stabilize ecosystems [8, 9]. By contrast, under severe drought, vapor pressure deficit (VPD) rises sharply, and warming‐amplified evaporative demand completely counteracts the water‐conserving effect of stomatal closure, accelerating soil moisture depletion and exacerbating drought stress [10, 11]. The above results indicate that the regulation of plant water relations by the increase of CO_2_ is not a single trend but presents a dual effect of “moderate relief exacerbated deterioration” with the change of stress intensity and atmospheric conditions.

**FIGURE 1:**
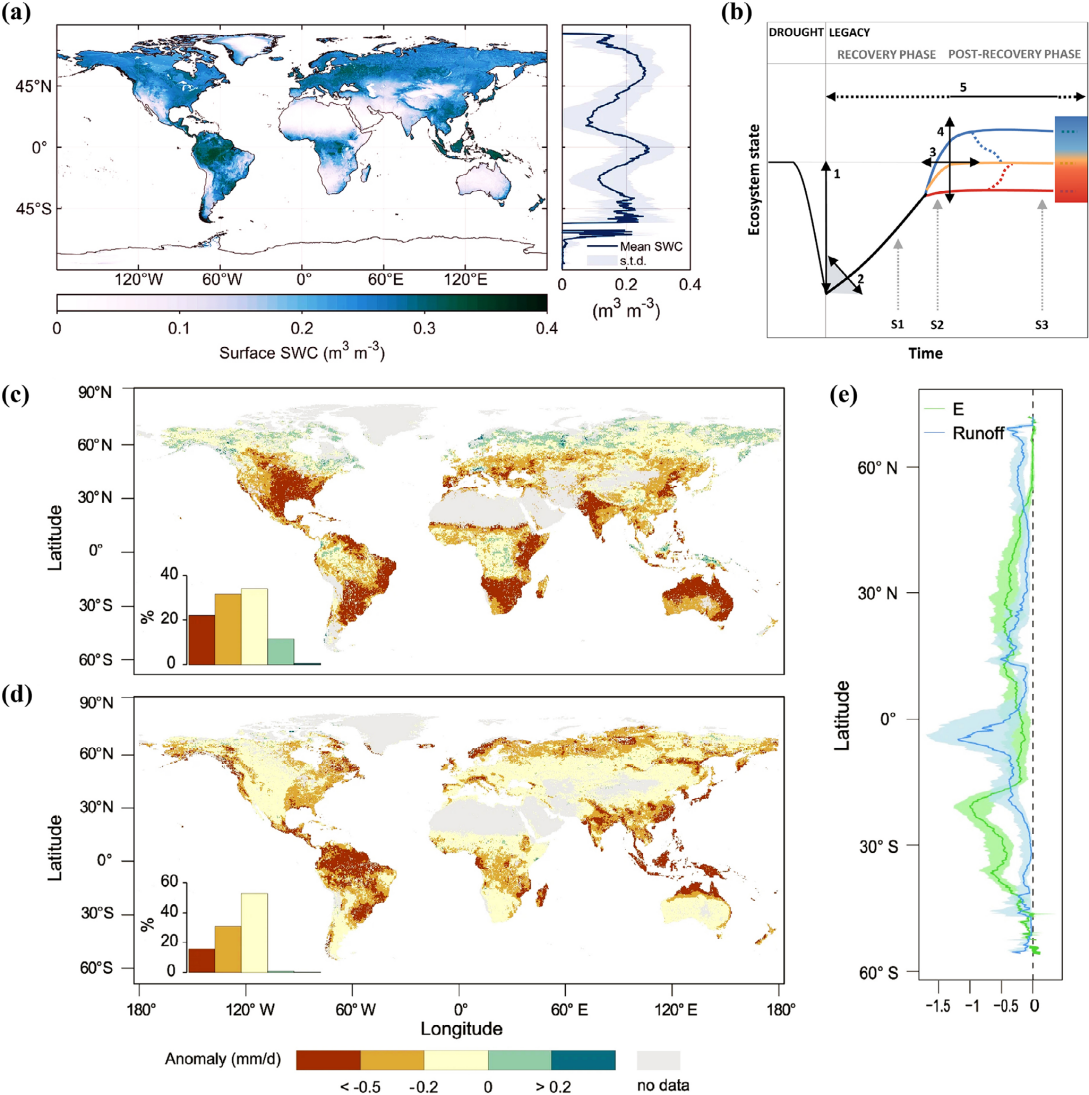
Global surface soil water dynamics, post‐drought recovery trajectories, and hydrological anomalies. (a) Annual mean surface soil water content (SWC) of SiTHv2 from 1982 to 2020. Adapted with permission from [24], Copyright 2024, Scientific Data; (b) The post – drought trajectories include the recovery and post – recovery phases. The recovery phase is marked by the recovery rate (2) after the drought's maximum impact (1). The post – recovery phase begins when the recovery rate is zero (3). This phase may show either complete recovery (yellow trajectory) or a shifted baseline indicating an immediate drought legacy (red and blue trajectories). In the post‐recovery phase, drought legacies are characterized by deviation from the pre‐drought baseline (4) and legacy duration (5). Reproduced with permission from [25], Copyright 2022, Global Change Biology; (c) Illustrates increased evapotranspiration (ET) in the high latitudes of the Northern Hemisphere and tropical regions during droughts, while subtropical and mid‐latitude regions experience a decrease. Adapted with permission from [13], Copyright 2025, Communications Earth & Environment; (d) Indicates reduced runoff across most of the globe. Adapted with permission from [13], Copyright 2025, Communications Earth & Environment; Adapted with permission from [13], Copyright 2025, Communications Earth & Environment; (e) Summary of the increase in ET at high latitudes and the tropics, the decrease in subtropical and mid‐latitude regions, and the overall reduction in global runoff. Adapted with permission from [14], Copyright 2025, Communications Earth & Environment.

The availability of water resources directly determines crop phenology and agricultural productivity. Soil water availability varies substantially across latitudes, the highest water content found in tropical and high latitude zones while the lowest soil water content areas are around 20 °N and 20 °S, where deserts dominate (Figure 1a). Therefore, climate change will differentially influence drought stress susceptibility between high‐and low‐latitude regions. Many subtropical regions are expected to become drier as the clime change progresses, leading to reduced vegetation cover, which in turn may enhance the expansion of deserts [12]. Drought‐prone areas are also ‐expected to increase in northern and southern Africa, southern Europe, Australia, and parts of Latin America (Figure 1c–e) [13]. Based on the analysis of hierarchical Bayesian models over the past 60 years, a study estimated that drought‐induced yield loss probability exceeds 90% across rain‐fed agricultural systems in East Africa and South Asia, with a long‐term average loss of approximately 14% [14]. By contrast, East Asia, which encompasses humid monsoon regions such as southern China, southern Japan, and southern South Korea, has demonstrated considerably stronger drought resistance and resilience. This enhanced resilience is largely attributable to well‐developed irrigation infrastructure and the availability of abundant germplasm resources of drought tolerant staple crops in the region [14].

Drought can have drastic impacts on the ecosystem as a whole, affecting plant growth and their microbial community [15, 16], while also affecting aboveground and belowground microbial community composition of the next‐generation of plants growing in drought impacted areas [17, 18]. This lag effect is also known as “drought memory” [19] or “drought legacy” (Figure 1b), which can severely weaken carbon sequestration in terrestrial ecosystems, further exacerbating the negative effects of climate change [20, 21]. The strength of the drought legacy effect depends on the length, intensity, and frequency of drought [22, 23].

Plant secondary metabolites (PSMs) constitute a chemically diverse group of compounds that play multifaceted roles in drought adaptation. Among these, flavonoids have emerged as a particularly prominent class in drought stress research, owing to their dual functions in antioxidant defense, stomatal regulation, and root system architecture remodeling [26]. Unlike terpenoids and alkaloids—other major classes of PSMs—flavonoids are distinguished by their rapid drought‐inducible biosynthesis, structural diversity that enables scavenging of multiple reactive oxygen species (ROS), and well‐characterized regulatory networks (e.g., the MYB‐bHLH‐WD40 transcriptional complex [27]), rendering them a tractable model system for dissecting plant adaptive mechanisms to water deficit. Accordingly, this review provides a systematic synthesis centered on flavonoids, with four specific objectives: 1) to survey current drought monitoring and prevention approaches and their applicability across agricultural systems; 2) to examine the impact of drought stress on plant secondary metabolism, with particular emphasis on flavonoid dynamics and their underlying regulatory circuits; 3) to explore how integrated agricultural practices—including irrigation regimes, soil amendments, cropping systems, and nanotechnological interventions—can modulate the plant microbiome to promote the release of beneficial secondary metabolites, thereby alleviating drought stress; and 4) to discuss the strategic selection of drought‐adapted varieties, the potential of hybridization, and other promising agronomic practices for enhancing crop resilience in arid regions.

## 2 Physiological Monitoring of Plant Drought Stress Responses

### 2.1 Physiological Indicators of Drought Stress

Plants have evolved diverse drought resistance strategies associated with morphological and physiological traits. Elucidating their mechanisms under extreme short‐term drought can provide a theoretical framework for predicting plant responses to climate change and a scientific basis for reducing drought‐related losses [28].

### 2.2 Indicators of Plant Water Status

Relative water content (RWC) and water potential are two important physiological indicators of drought stress in plants. RWC reflects the moisture retention capacity of plant tissue under stress conditions [29], calculated by comparing fresh weight, saturated weight, and dry weight of plant tissue, usually expressed as percentages. In turn, water potential reflects the free energy state and movement of water in plant cells [30], usually measured in megapascals (MPa), and can be measured in a pressure chamber or with a dew point water potential meter. Under drought stress, drought‐tolerant varieties usually have higher RWC values and more stable water potential, which may be due to their stronger moisture absorption and lower stomatal moisture loss. Although both RWC and water potential are related to the plant ‘s moisture state, water potential more directly reflects the free energy and moving force of water, while RWC reflects the moisture retention ability of plant tissue [31]. Therefore, both RWC and water potential can be used as important indicators for evaluating the health of plants under drought stress. A study subjected 10 maize (*Zea mays* L.) lines, including drought‐tolerant and drought‐sensitive genotypes, to cyclic drought stress and rewatering treatments [32]. The results revealed that drought stress significantly reduced leaf RWC and leaf water potential across all genotypes, yet the drought‐tolerant lines maintained superior water status, with a leaf relative water content of 64.3% and a leaf water potential of −1.59 MPa under drought conditions. These tolerant genotypes also showed rapid recovery of drought‐related physiological indicators following rewatering, confirming that leaf RWC and leaf water potential serve as reliable physiological markers for screening drought‐adapted maize (*Zea mays* L.) genotypes [32]. Genome‐wide association research investigating the molecular regulatory pathways underlying leaf RWC and drought tolerance in maize (*Zea mays* L.) has identified the key functional allele *ZmSRO1d‐R*. This allele enhances the mono‐ADP‐ribosyltransferase activity acting on *ZmRBOHC*, elevating reactive oxygen species (ROS) levels in guard cells, accelerating stomatal closure and reducing transpiration water loss. As a result, maize maintains higher leaf RWC under drought stress, improving overall drought resistance and stabilizing grain yield [33]. Guard cell ROS serve as a core signaling hub linking stomatal movement and flavonoid metabolism. On the one hand, abscisic acid (ABA)‐triggered ROS bursts act as the central signal to initiate stomatal closure; on the other hand, flavonols specifically accumulated in guard cells function as non‐enzymatic antioxidants to mitigate excessive ROS, preventing extreme stomatal closure that would impair photosynthesis. Meanwhile, drought‐induced ROS signals upregulate key flavonoid biosynthetic genes including chalcone synthase (CHS) and flavonol synthase (FLS), facilitating endogenous flavonoid accumulation and establishing a negative feedback regulatory loop [34]. This loop not only alleviates membrane lipid peroxidation but also dynamically balances stomatal water loss with photosynthetic carbon assimilation efficiency. As highly efficient non‐enzymatic antioxidants, flavonoids directly scavenge excess ROS, mitigate oxidative stress, and fine‐tune stomatal behavior—a mechanism that has been experimentally validated in the drought response of maize (*Zea mays* L.) seedlings [35].

### 2.3 Photosynthetic Function Parameters: Chlorophyll Fluorescence (Fv/Fm)

In addition to RWC and water potential, chlorophyll fluorescence parameters—particularly the maximum photochemical efficiency of photosystem II (PSII), denoted as Fv/Fm—together with membrane stability index (MSI), commonly assessed via relative electrical conductivity (REC), have become widely adopted physiological indicators for evaluating plant drought tolerance [36]. The Fv/Fm ratio reflects the intrinsic photochemical efficiency of PSII and is highly sensitive to environmental stressors including drought. Under drought stress, impairment of PSII reaction centers and disruption of electron transport chains lead to a decline in Fv/Fm [37]; notably, this parameter remains relatively stable under mild stress but decreases markedly under severe drought, rendering it a sensitive diagnostic tool for PSII photoinhibition and overall photosynthetic performance, as well as an effective physiological marker for screening drought‐tolerant germplasm. For instance, drought‐tolerant barley (*Hordeum vulgare* L.) genotypes exhibited significantly smaller reductions in Fv/Fm under drought stress compared to sensitive genotypes, indicating milder PSII photoinhibition and superior photoprotective or repair capacity [38].

### 2.4 Photosynthetic Structural Parameters: Chlorophyll and Carotenoid Contents

Chlorophyll content serves as a key indicator of drought stress severity and plant adaptive capacity, with its dynamic changes directly reflecting physiological status and stress intensity. Under drought conditions, chloroplasts—being the primary sites of ROS generation—are among the first organelles to suffer oxidative damage [39]. Excessive ROS accumulation degrades photosynthetic pigments on thylakoid membranes, leading to a general decline in chlorophyll content under water deficit [40]. Consequently, genotypes capable of maintaining higher chlorophyll a (Chl a) levels under drought, or plants treated with antioxidant biostimulants, typically exhibit superior photosynthetic recovery capacity and enhanced drought tolerance.

Quantitative evidence from gradient drought experiments in dryland maize (*Zea mays* L.) has established a numerical relationship between chlorophyll retention and overall drought resilience. In a controlled study on maize (*Zea mays* L.) cultivar ‘Zhengdan No. 16’ subjected to four water regimes ranging from well‐watered to severe drought, exogenous application of ascorbic acid (AA) increased leaf Chl a content by 11.0%, while salicylic acid (SA) treatment resulted in a 14.5% increase relative to untreated controls under well‐watered conditions. Under severe drought stress, combined AA+SA application enhanced carotenoid content by 14.3%, significantly alleviating drought‐induced Chl a degradation and improving pigment retention under extreme water deficit [41]. Field‐based evidence further substantiates this quantitative relationship. In maize (*Zea mays* L.) subjected to 40% field capacity, drought stress alone reduced total chlorophyll by 15% and carotenoids by 19%, whereas foliar application of AA (100 mg/L) and SA (100 mg/L) in combination increased total chlorophyll by 47% and carotenoids by 43% compared to untreated drought‐stressed plants [42]. This pigment retention effect was closely associated with the coordinated activation of antioxidant defense systems—superoxide dismutase (SOD) and catalase (CAT) activities increased by 45% and 38%, respectively, while malondialdehyde (MDA) and hydrogen peroxide (H_2_O_2_) accumulation decreased by 36% and 32%, respectively. Concurrently, osmotic adjustment capacity was enhanced, as reflected by 46% and 20% increases in proline and soluble protein contents [42]. Collectively, these quantitative data demonstrate, across three interconnected dimensions—antioxidant coordination, osmotic regulation, and pigment stabilization—that the extent of photosynthetic pigment retention under drought serves as a reliable indicator of drought tolerance in staple crops.

Beyond their role as photosynthetic pigments, carotenoids fulfill dual photoprotective and signaling functions. On one hand, carotenoids protect chlorophyll from photo‐oxidative damage by quenching excess excitation energy and scavenging ROS [43]. On the other hand, photo‐oxidative stress triggers the accumulation of carotenoid‐derived signaling molecules—such as β‐cyclocitral and β‐ionone—which systematically modulate downstream gene expression, promoting strigolactone biosynthesis to regulate lateral branch development and facilitating arbuscular mycorrhizal fungi (AMF) colonization [44, 45]. Strigolactones, as carotenoid‐derived phytohormones, play a pivotal role in drought responses. In tomato (*Solanum lycopersicum* L.), silencing the strigolactone biosynthesis gene *CCD7* compromises plant drought resistance by impairing stomatal closure and diminishing antioxidant defense system activity [46]. Conversely, exogenous application of the strigolactone analog *GR24* restores root development under drought through modulating polar auxin transport [47]. Beyond their well‐established functions in stomatal regulation and root architecture remodeling, strigolactone signaling is functionally intertwined with flavonoid biosynthesis, forming a coordinated regulatory network that synergistically enhances drought tolerance. At the transcriptional level, SL signaling converges with the flavonoid pathway via the *MAX2‐BES1* regulatory module. *BES1* functions as a transcriptional repressor of key *MYB* transcription factors—*MYB11*, *MYB12*, and *MYB111*—that activate flavonoid structural genes, including *TT4*, *TT5*, *F3H*, and *FLS*. *MAX2*‐mediated degradation of BES1 relieves this repression, thereby promoting flavonoid accumulation [48]. This regulatory link is further substantiated by metabolomic and transcriptomic evidence. In drought‐stressed soybean (*Glycine max*), foliar application of SLs significantly upregulated the flavone and flavonols biosynthesis pathway [49].

### 2.5 Cellular Integrity Parameters: Membrane Stability

Parallel to chlorophyll fluorescence, membrane stability serves as a critical indicator of cellular integrity under drought stress. Drought‐induced oxidative damage compromises the structural integrity of the plasma membrane, resulting in increased permeability and subsequent leakage of electrolytes and solutes [50]. This damage is quantitatively assessed through REC measurements, with higher REC values indicating greater membrane injury. Drought‐tolerant genotypes typically maintain lower REC under water‐deficit conditions, reflecting superior membrane stability and enhanced capacity to preserve cellular compartmentalization. For example, In a large‑scale study, leaf tissues from 2111 spring wheat (*Triticum aestivum* L.) accessions were subjected to heat and PEG‑induced osmotic stress to evaluate cell membrane stability (CMS), followed by genome‑wide association analysis [51]. The results revealed highly significant CMS variation among accessions and identified multiple single nucleotide polymorphisms (SNPs) strongly associated with CMS, most of which were located in genomic regions implicated in transmembrane ion transport and abiotic stress responses. Preliminary field validation further indicated that genotypes with superior CMS tended to achieve better yield performance. A separate screening study of 25 rice (*Oryza sativa* L.) cultivars under drought conditions verified that two tolerant varieties (GAR‐13 and NWGR‐16026) possessed higher membrane stability index (MSI), supporting MSI as a robust physiological marker for drought tolerance screening in rice (*Oryza sativa* L.) [52].

### 2.6 Osmotic Adjustment and Antioxidant Protection: Proline

Plants can also alleviate drought stress through the accumulation of solutes in cells to maintain the osmotic balance and cell structure, and to activate the antioxidant system to reduce water loss [53]. Proline is a key osmoprotectant in plants [54] and is widely used as an indicator of stress tolerance. Under drought conditions, plants enhance stress resistance by increasing proline accumulation in tissues [55, 56] and by upregulating the activity of key enzymes in the proline biosynthesis pathway, such as pyrroline‐5‐carboxylic acid synthase (P5CS) [57]. Exogenous application of proline can effectively improve crop drought resilience. Beyond its role as an osmoprotectant, proline also functions as a signaling molecule that modulates secondary metabolism. Specifically, proline accumulation helps stabilize intracellular redox homeostasis, thereby alleviating ROS‐induced damage to the nuclear transcriptional machinery and transcriptionally activating key flavonoid biosynthetic genes—including *chalcone synthase* (CHS) and *flavonol synthase* (FLS)—to sustain endogenous flavonoid production. Conversely, insufficient flavonoid biosynthesis exacerbates cellular oxidative stress, which in turn upregulates *P5CS* expression and triggers compensatory proline accumulation [54], forming a regulatory feedback loop that maintains redox balance. This functional interplay between proline and flavonoid pathways is further supported by genetic evidence: the soybean (*Glycine max*) *R2R3‐MYB* transcription factor *GmMYB12* has been shown to coordinately upregulate genes involved in both proline biosynthesis (e.g., *P5CS*) and flavonoid accumulation, thereby enhancing drought tolerance in transgenic plants [58]. One study demonstrated that a single foliar spray of 1 mM proline before drought stress creates a long‐term priming effect on winter wheat (*Triticum aestivum* L.) drought tolerance [59]. A 35‐day proline pre‐treatment induced drought stress memory, enhanced osmotic adjustment, protected photosystem II function, and reduced membrane injury under subsequent drought stress, while facilitating the rapid recovery of photosynthesis and transpiration after rewatering. These results confirm that exogenous proline improves both drought resilience and post‐drought recovery performance [59].

### 2.7 Drought memory

**FIGURE 2:**
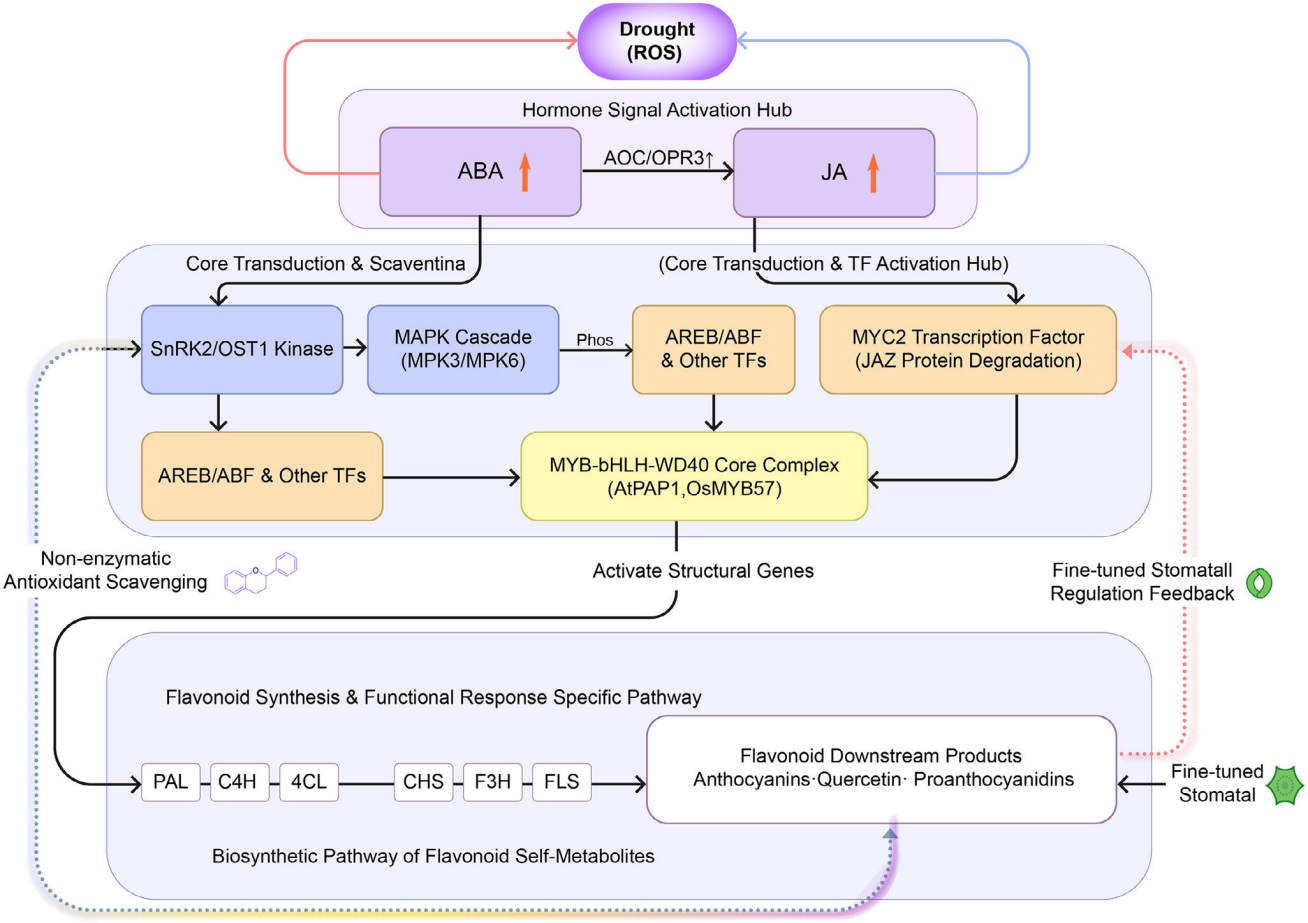
Regulatory cascade linking drought‐triggered ROS, ABA/JA phytohormone signaling and flavonoid biosynthesis in crop drought resistance.

“Drought memory” refers to the morphological, molecular, and physiological changes that plants undergo during an initial drought event—such as reduced leaf number, plant height, and biomass [60, 61, 62]—which enhance their adaptability to subsequent drought episodes [63]. These adaptive responses are modulated by alterations in root water status and shifts in soil microbial communities [64]. Plants reinforce drought tolerance through signaling molecules [65], phytohormones, and secondary metabolites that regulate stress adaptation and survival under water deficit [65, 66]. Key phytohormones—including ABA, ethylene, jasmonic acid (JA), and auxins—play central roles in drought responses. ABA regulates stomatal closure and root development [67]; ethylene and JA enhance drought and cold tolerance [68], and auxins modulate root system architecture [69]. Notably, ABA and JA signaling are functionally interconnected in drought responses: ABA activates the expression of JA biosynthesis genes, such as *AOC* and *OPR3*, thereby promoting JA accumulation and reinforcing drought resistance [68]. As visualized in our schematic diagram (Figure 2), the crosstalk between ABA and JA further triggers a sequential kinase cascade and MYB‐bHLH‐WD40 transcription complex, which ultimately activates the entire flavonoid biosynthetic pathway to eliminate drought‐induced ROS and form a feedback regulatory loop for stomatal movement. Additionally, ABA induces the production of dehydrins—protective proteins that help maintain cellular structural integrity and function under drought conditions, mitigate water‐loss‐induced damage, and enhance overall drought resilience [70]. Beyond phytohormonal and metabolic regulation, drought memory is increasingly recognized as being underpinned by epigenetic mechanisms—heritable changes in gene expression that occur without alterations in the underlying DNA sequence [49]. These mechanisms, including DNA methylation, histone modifications, and non‐coding RNAs, enable plants to retain information from prior drought exposure and mount faster, more robust responses upon subsequent encounters [71]. DNA methylation dynamically reshapes chromatin landscapes in response to drought. Stress‐induced DNA demethylation occurs near drought‐responsive genes, and specific methylation patterns can be inherited by progeny, conferring enhanced drought adaptability. In rice (*Oryza sativa* L.), natural epialleles such as *DRO1* demethylation enhance drought adaptation through deeper rooting, providing 15%–22% yield protection. Histone modifications serve as stable epigenetic marks that encode stress memory. In Arabidopsis subjected to recurring dehydration stress, a subset of stress‐responsive genes retains atypically high levels of H3K4me3 (trimethylation of histone H3 at lysine 4) and stalled RNA polymerase II during recovery intervals [72]. This stalled polymerase can be rapidly reactivated by pause‐release factors upon subsequent drought exposure, enabling accelerated transcriptional induction—a mechanism termed transcriptional memory, representing the first documented example of stalled RNA polymerase II involvement in plant stress memory [72, 73]. In maize (*Zea mays* L.), histone acetylation coordinates with DNA methylation to orchestrate stress‐responsive pathways, ensuring rapid adaptation to recurrent drought [74]. The hierarchy of somatic stress memory is characterized by sustained H3K4me3 enrichment at promoters, which facilitates rapid re‐induction upon stress re‐exposure [75]. Non‐coding RNAs fine‐tune chromatin states to reinforce drought memory. Small RNAs act as critical regulators by altering transcript levels of drought‐responsive target genes, while long non‐coding RNAs—such as DANA2 in Arabidopsis—positively regulate drought tolerance by modulating chromatin enrichment at key loci [73]. Beyond these somatic mechanisms, transgenerational epigenetic inheritance enables drought‐induced epigenetic imprints to be transmitted from parent to offspring via RNA‐directed DNA methylation (RdDM) across successive generations, thereby priming progeny for recurrent stress challenges [72]. However, prolonged recovery intervals can lead to the loss of drought memory through the action of specific demethylases and chromatin accessibility factors [73]. Collectively, these epigenetic mechanisms, including DNA methylation, histone modifications, and non‐coding RNAs, operate in concert with hormonal signaling to establish both short‐term and transgenerational drought memory, providing a molecular basis for the enhanced resilience observed in primed plants.

Due to their importance in plant's drought response, plant hormone levels can be used as a measure of drought memory via HPLC or GC‐MS, analyzing gene expression of drought‐responsive elements via qRT‐PCR or RNA‐seq methods, and by detecting signaling molecules like ROS and NO using fluorescence microscopy or chemiluminescence assays [76, 77]. These techniques provide insights into the molecular mechanisms underlying drought memory and identify targets for improving plant resilience and are commonly used in plant drought stress research. However, these methods may not be practical for farmers and other food producers, who often monitor the effects of drought stress by observing morphological changes in plants. For example, curled leaves, wilting or yellowing of crops are common signs of drought stress, and these characteristics can help farmers determine whether crops are dehydrated. However, these visual signs are not exclusive to drought stress and may also occur during other abiotic or biotic stresses. Some agricultural services offer diagnostic tools based on plant morphology and growth status to help farmers identify the nutrient needs of crops and guide irrigation and fertilization. These morphology‐based monitoring methods, while relatively simple, are practical and straightforward for farmers to monitor drought responses.

Plant breeders also employ a variety of different methods when selecting drought‐resistant varieties. While they may not directly measure hormone or RNA levels, they use indirect indicators to assess the drought resistance of plants. For instance, breeders observe the growth performance of plants under drought conditions, including morphological and physiological indicators such as plant height, number of leaves, root development, and seed yield. Furthermore, breeders conduct field trials and drought tolerance screening tests by planting different crop varieties under drought stress to evaluate their survival rate, growth rate, and yield loss. Although these methods do not involve complex molecular biology techniques, they effectively screen for more drought‐resistant varieties, providing options better adapted to drought environments for agricultural production. Nevertheless, traditional phenotypic screening still serves as a reliable preliminary method to identify drought‐resilient varieties, yet thorough elucidation of core molecular mechanisms such as hormone signal transduction and gene regulatory networks facilitates the application of precise genetic editing, greatly shortening the breeding cycle of highly drought‐tolerant new cultivars.

## 3 Plant Secondary Metabolites and Their Role in Drought Resistance

Drought poses a severe threat to ecosystems and has a significant negative impact on the physiological processes of plants [78, 79]. Plants are capable of producing thousands of low molecular weight organic compounds, which can be categorized based on their functions and properties into plant hormones, primary metabolites, and secondary metabolites. Plant hormones regulate plant growth, development, and responses to the environment, and are involved in signal transduction [80]. Primary metabolites (such as amino acids and lipids) directly participate in essential physiological processes and cellular structure composition, whereas secondary metabolites (such as terpenoids and phenolics) primarily regulate biological processes and metabolism [81]. Flavonoids constitute one of the major categories of phenolic specialized metabolites and are traditionally classified into several subclasses based on the oxidation state and substitution pattern of their central C‐ring [82]. These include flavones (e.g., apigenin, luteolin), flavonols (e.g., quercetin, kaempferol), flavanones (e.g., naringenin, hesperetin), flavanols/catechins (e.g., catechin, epicatechin), isoflavones (e.g., daidzein, genistein), and anthocyanins (e.g., cyanidin, delphinidin) [83]. Among these subclasses, flavonols and anthocyanins have been extensively investigated for their robust ROS‐scavenging capacity under drought conditions. Of particular interest, a study on purple corn (*ZEA MAYZ* L.) revealed that C‐glycosyl flavone‐enriched extracts can form copigmentation complexes with anthocyanins (cyanidin and pelargonidin derivatives), substantially broadening the color spectrum and enhancing pigment stability, with half‐lives extended by up to 50% [84]. This copigmentation effect prolongs the functional persistence of antioxidant metabolites. Notably, both the anthocyanidin type and acylation status emerged as critical determinants of stability [84]. Meanwhile, flavanones and flavones function as key intermediates in stress‐triggered metabolic reprogramming or serve as bioactive end products [85]. Taking wheat (*Triticum aestivum* L.) as an example [86], integrated transcriptomic and metabolomic analyses demonstrated that drought‐tolerant seedlings (T13) significantly activate the flavone and phenolic acid biosynthetic pathways under drought stress, with key genes including *HCT*, *FLS*, *CHS*, and *F3′5′H* being substantially up‐regulated. In contrast, drought‐sensitive seedlings (T2) preferentially accumulated alkaloids. This metabolic divergence points to the flavonoid‐phenolic acid module as a central metabolic signature of drought tolerance in wheat, thereby offering actionable targets for molecular breeding [86]. Collectively, these three categories of compounds—phytohormones, primary metabolites, and secondary metabolites—play indispensable roles in plant growth and development, interspecific competition and defense, as well as environmental adaptation [87].

The biosynthetic pathways of PSMs are highly branched, typically occurring in specific organs, tissues, cells, or organelles, and are independently regulated, possessing various ecological functions [88, 89]. The types of secondary metabolites mainly include nitrogen‐containing organic compounds, terpenoids, and phenolic compounds. These metabolites are the result of plants' adaptation to abiotic stresses such as drought, temperature, and radiation, as well as to biotic stresses such as pathogen infection and herbivory, and have significant impacts on the relationship between plants and their environment by influencing plant growth, defense mechanisms, and interactions with other organisms [90]. When plants are infected by pathogenic microorganisms, they massively synthesize and accumulate secondary metabolites to enhance immunity and disease resistance [91]. Under drought stress conditions, plants accumulate secondary metabolites with antioxidant activity to assist in the synthesis and transport of metabolites and enzymes, stabilize cellular components, and participate in signal transduction and gene regulation [92]. Additionally, the synthesis, accumulation, and transport of secondary metabolites are induced by environmental factors, exhibiting specificity depending on plant tissue, developmental stage, and the type of environmental stress. For instance, when subjected to abiotic stresses such as drought, high temperature, and radiation, plants synthesize flavonoids to consume excess triose phosphate, ATP, and NADPH, forming an energy safety valve mechanism that alleviates stress damage [78, 79]. This mechanism not only helps plants maintain physiological stability under stress conditions but also provides a critical metabolic regulatory strategy for plants to adapt to harsh environments.

Temperature and drought jointly affect soil phosphorus (P) availability, with flavonoids serving as the core molecular mediators connecting rhizosphere P transformation and crop drought adaptability. Elevated temperature promotes root exudation of flavonols, which enhance P mobilization and recruit beneficial rhizosphere microorganisms to accelerate organic P mineralization, thereby alleviating low‐P stress [93]. Conversely, drought reduces available inorganic P and increases insoluble P forms, further inhibiting root growth and organic P mineralization [94, 95], establishing a vicious cycle of “P immobilization—root restriction—reduced exudation” that exacerbates drought damage. At the molecular level, drought‐induced ABA signaling and P‐starvation signaling synergistically activate the PHR1–WRKY33–MBW regulatory module: PHR1 interacts with the MBW complex to induce the expression of flavonoid biosynthetic genes such a *CHS* and *F3'H*, while simultaneously relieving WRKY33‐mediated transcriptional repression of *DFR*, forming a double‐negative logic switch that dynamically modulates flavonoid accumulation in response to P status [96, 97]. The accumulated flavonoids, on one hand, directly scavenge drought‐triggered ROS, protect root meristem activity and promote lateral root development, thereby expanding P uptake area under water‐limited conditions; on the other hand, they feed back to improve the rhizosphere P environment through root exudation [98]. A study showed that drought significantly reduces P uptake and biomass accumulation in maize (*Zea mays* L.) [99], slows the replenishment of inorganic phosphorus in soil solution, and increases the proportion of poorly soluble calcium‐bound phosphorus to 45% [99]. Drought‐induced increases in soil pH and calcium content further convert phosphorus into insoluble, unavailable forms, drastically reducing overall soil phosphorus bioavailability. Therefore, in arid regions, rational fertilization and soil management practices not only directly improve soil physicochemical properties and enhance P availability but also alleviate root water deficits. Concurrently, these practices promote the sustained expression of key flavonoid biosynthesis genes in root epidermal cells, thereby mitigating both P deficiency stress and drought‐induced oxidative damage, and ultimately improving crop growth [100, 101, 102, 103].

### 3.1 Structure, Synthesis and Ecological Functions of Flavonoids for Plants

**FIGURE 3:**
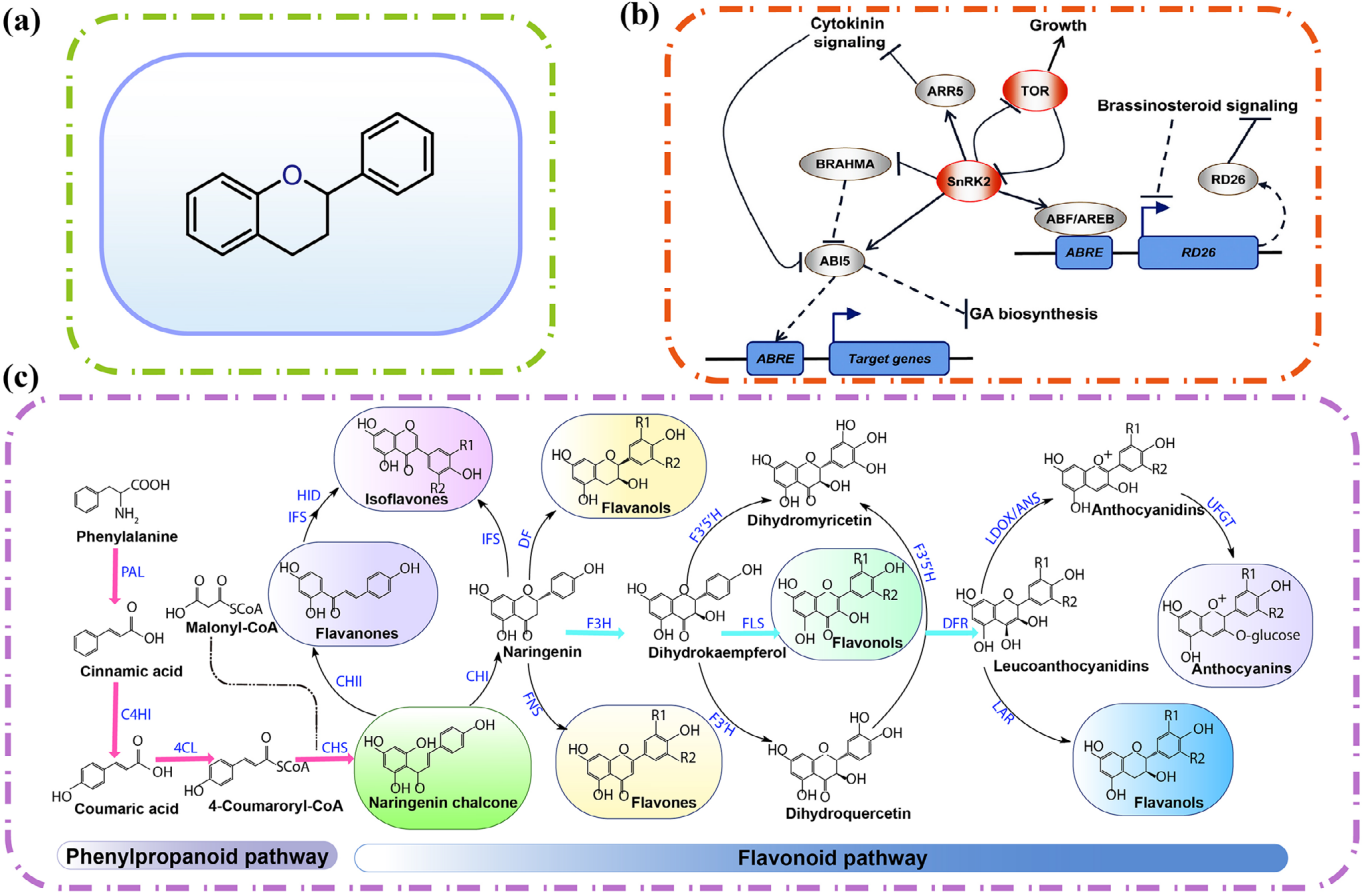
Flavonoid structure, hormone‐mediated stress regulation, and biosynthetic pathways. (a) Flavonoids structural skeleton; (b) Figure b illustrates the plant hormone signaling system's control over responses to abiotic stress through transcriptional regulation, particularly the interaction of the abscisic acid (ABA) signaling pathway with other hormone pathways during transcription. Under drought stress, AREBs/ABFs activate the transcription of drought‐responsive genes (e.g., RD26). Adapted with permission from [116], Copyright 2022, Nature Reviews Molecular Cell Biology.; (c) Biosynthetic pathways of flavonoids. Adapted with permission from [117], Copyright 2023, Molecules.

Flavonoids constitute the largest category of plant phenolic compounds, with over 10 000 individual compounds identified to date [104]. Structurally, they are characterized by a typical C6‐C3‐C6 skeleton [105], consisting of two aromatic rings (A and B) linked by a central three‐carbon chain (Figure 3a). Based on the oxidation state and substitution pattern of the central C‐ring, flavonoids are traditionally classified into several major subclasses, including flavonols (e.g., quercetin, kaempferol), flavones (e.g., apigenin, luteolin), flavanones (e.g., naringenin, hesperetin), flavanols/catechins (e.g., catechin, epicatechin), isoflavones (e.g., daidzein, genistein), and anthocyanins (e.g., cyanidin, delphinidin) [83]. Compounds within the same subclass are further distinguished by variations in A‐ and B‐ring substituents, such as hydroxylation, methylation, and glycosylation [106] (Figure 3c). These naturally occurring bioactive compounds serve as key ingredients in pharmaceuticals, nutraceuticals, cosmetics, and other commercial applications (Table 1).

**TABLE 1:** Structural and functional analysis of common flavonoid compounds.

Flavonoids	Structure	Molecular formula	Activity	Ecological Function	References
Cyanidin Chloride	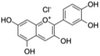	C_15_H_11_ClO_6_	Antioxidant, anti‐cancer, natural pigments	Absorb UV radiation	[129]
Isoflavone	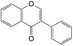	C_15_H_10_O_2_	defend, regulate plant growth, prevent cardiovascular and cerebrovascular, anti – cancer	Possess allelopathic effects	[130, 131, 132]
flavanones	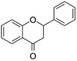	C_15_H_12_O_2_	Antioxidant, anti‐inflammatory	Promote plant‐microbe Symbiosis	[133, 134]
Hesperidin	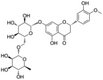	C_28_H_34_O_15_	Protect cardiovascular system, treat obesity, treat Diabetes	Reducing soil heavy metals	[135, 136, 137]
Luteolin	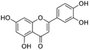	C_15_H_10_O_6_	anti‐inflammatory, anti‐tumor, anti‐diabetic, antioxidant	Degradation of environmental pollutants (DEHP)	[138, 139]
Kaempferol	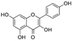	C_15_H_10_O_6_	Alleviate the oxidative stress damage of the retina, anti‐inflammatory, anti‐hyperglycemic	Reduce Pb accumulation	[140, 141, 142]
Quercetin	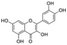	C_15_H_10_O_7_	Antioxidation, neuroprotection, anticancer	Reducing salt and heavy metal stress	[143, 144]
Baicalin	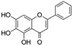	C_15_H_10_O_5_	Anti‐inflammatory, antibacterial, antitumor.	Recruit bacteria that promote rhizosphere growth and enhance soil nutrients	[145, 146]
Rutin	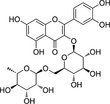	C_27_H_30_O_16_	Anti‐diabetes, antioxidation, anti‐aging, nerve protection	Mitigating Plant Drought Stress	[147, 148, 149, 150]
Arbutin	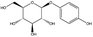	C_12_H_16_O_7_	Antioxidant, anti‐inflammatory, anti‐tumor, inhibiting tyrosinase.	Interaction with microorganisms promotes plant growth.	[151, 152]

Flavonoids have multiple roles for plants, functioning for example, as ultraviolet (UV) filters, enhancing nutrient acquisition and aid defenses against biotic stress [107, 108], thus serving as a critical factor in the successful “land colonization” of plants [109]. Abiotic stress, such drought and high light intensity impact flavonoid production which together with other phenolic compounds play an important role plant's stress tolerance [80, 110]. Flavonoids are also key metabolites involved in plant drought tolerance and participate in drought stress responses through a series of reactions including signal transduction, gene regulation, ROS scavenging, and stomatal movement [111]. For example, the accumulation of catechins in tea (*Camellia sinensis* (L.) O. Kuntze) significantly increases under drought conditions, helping to neutralize free radicals and maintain cellular physiological functions [112]. The expression of flavonoid biosynthesis pathways is often upregulated during drought stress together with other drought‐response genes [113, 114]. In peanut (*Arachis hypogaea* L.), the accumulation patterns of phenols, flavonols, and anthocyanins were examined across six varieties under water deficit conditions; the BARI2011 variety exhibited higher water retention and flavonoid accumulation, contributing to the maintenance of normal physiological balance [115]. In maize (*Zea mays* L.), the drought‑tolerant mutant *doi57* outperformed the wild‑type B73 under drought conditions, a phenotype likely attributable to the upregulated expression of flavonoid biosynthesis‑related genes [35]. Under water deficit, guard cells of *doi57* accumulated more flavonols and less H_2_O_2_, and its seedling extracts displayed stronger oxygen free‑radical scavenging activity [35].

Flavonoids also protect photosynthetic pigments from oxidative damage and help maintain normal photosynthesis. Under drought stress, flavonoids can effectively quench singlet oxygen produced in chloroplasts, protecting the thylakoid membrane and the outer chloroplast membrane from lipid peroxidation [83]. Flavonoids can also regulate stomatal opening and closure to reduce water evaporation and maintain plant water balance. Under drought conditions, flavonoids can inhibit the production of H_2_O_2_ induced by ABA in guard cells, thereby preventing stomatal closure and maintaining higher stomatal conductance and photosynthetic rates. In Arabidopsis thaliana, exogenous quercetin treatment was observed to attenuate ABAinduced stomatal closure, suggesting that quercetin may counteract ABA signaling by scavenging the ROS responsible for stomatal closure [118]. Conversely, the Arabidopsis chalcone synthase mutant *tt42*, which is defective in flavonol synthesis, exhibited a hypersensitive response to ABA, with more pronounced stomatal closure compared with the wild type [118]. However, the role of flavonoids in regulating stomatal movement is more complex and species‐dependent than previously assumed. In the widely adopted model derived from Arabidopsis thaliana, flavonols function as ROS scavengers in guard cells, attenuating ABA‐induced ROS bursts and thereby inhibiting stomatal closure [118]. This paradigm is supported by evidence from tomato (*Solanum lycopersicum* L.) [119], where the JA‐responsive transcription factor *SlMYC2* directly represses *SlCHS1* expression, decreasing flavonol accumulation, increasing ROS content in guard cells, and promoting stomatal closure under drought stress. Silencing *SlCHS1* produced a phenotype similar to that of *SlMYC2*‐overexpressing lines, confirming that *SlMYC2* drives stomatal closure by modulating flavonol accumulation.

Contrasting evidence from other species, however, challenges the universality of this model. In peanut (*Arachis hypogaea* L.) [120], exogenous quercetin or kaempferol significantly reduces ROS accumulation in guard cells while simultaneously promoting stomatal closure under drought stress. Mechanistically, exogenous flavonols upregulate the transcription of *AhNCED*1—the gene encoding the rate‐limiting enzyme in ABA biosynthesis—thereby stimulating ABA accumulation and activating stomatal closure signaling. This positions flavonols upstream of ABA signaling in peanut, acting as positive regulators of ABA production rather than downstream ROS scavengers. In tobacco (*Nicotiana tabacum* L.) [121], the *R2R3‐MYB* transcription factor *NtMYB184*, which belongs to the flavonol‐specific *SG7* subgroup, regulates flavonol biosynthesis in guard cells to modulate ROS homeostasis and stomatal aperture. ABA‐induced ROS production is accompanied by suppression of *NtMYB184* and flavonol biosynthesis, which may accelerate ABA‐induced stomatal closure. By contrast, ethylene stimulates *NtMYB184* expression and flavonol biosynthesis to suppress ROS accumulation and curb ABA‐induced stomatal closure. In *MYB184* mutants, neither flavonol and ROS concentrations nor stomatal aperture varied between ABA and ABA+ethylene treatments, indicating that *NtMYB184* is indispensable for the antagonism between ethylene and ABA via regulating flavonol and ROS concentrations in guard cells [121]. These divergent observations demonstrate that the regulatory effects of flavonoids on stomatal movement are species‐specific, determined by their functional sites within the ABA signaling cascade, and simultaneously modulated by multiple factors including flavonol subtypes, glycosylation modifications, tissue distribution, and phytohormone crosstalk.

The orchestration of the aforementioned drought‐resilience mechanisms fundamentally relies on the stress‐responsive activation of flavonoid biosynthetic pathways. By chelating transition metal ions and modulating antioxidant enzyme kinetics, these polyphenolic compounds efficiently suppress ROS accumulation and fortify cellular defenses. The underlying biosynthetic framework is structured as a two‐tiered hierarchical network. It initiates with the core phenylpropanoid pathway, which synthesizes universal intermediates by converting phenylalanine to p‐coumaroyl‐CoA through the sequential actions of *PAL*, *C4H*, and *4CL* [122]. Diverging from this precursor, the downstream flavonoid‐specific branch drives metabolic flux toward flavonol production via sequential catalytic reactions mediated by *CHS*, *CHI*, *F3H*, and *FLS* [123, 124]. Drought stress drastically upregulates these structural genes, shifting metabolic prioritization toward flavonol accumulation. At the transcriptional level, this metabolic redirection is primarily governed by *R2R3‐MYB* transcription factors, including *MYB11/PFG2*, *MYB12/PFG1*, and *MYB111/PFG3* [125, 126], which directly bind and transactivate the promoters of CHS, FLS, and associated structural genes. However, to mitigate potential energetic costs and optimize carbon allocation, this transcriptional drive is tightly coupled with post‐transcriptional and post‐translational buffering systems. At the post‐transcriptional layer, specific microRNAs—most notably miR858 and *miR828*—operate as riboregulatory switches that induce endonucleolytic cleavage or translational inhibition of *R2R3‐MYB* transcripts, thereby ensuring that flavonol production remains dynamically proportional to the severity of water deficit [127]. Simultaneously, post‐translational modifications, including phosphorylation and ubiquitin‐proteasome system (UPS)‐mediated proteolysis, precisely shape flavonoid homeostasis. The operational lifespan and catalytic turnover of both upstream MYB activators and key downstream enzymes (such as *PAL* and *CHS*) are strictly regulated by specific E3 ubiquitin ligases, granting the metabolic reprogramming high reversibility and kinetic agility [128]. In conclusion, this multi‐tiered regulatory architecture, encompassing gene transactivation, microRNA‐mediated silencing, and UPS‐driven proteolysis, forms a cohesive and highly responsive drought‐adaptation network. This integrated paradigm offers a comprehensive molecular explanation for the heightened flavonol deposition in the *doi57* mutant as well as the hypersensitive stomatal behavior of the *tt4‐2* mutant.

Drought‐induced flavonoid accumulation is coordinated by multiple interconnected signaling pathways. Among these, the ABA dependent cascade serves as the central hub of the regulatory network: the AREB/ABF transcription factor family directly activates flavonoid biosynthetic genes, thereby promoting flavonoid enrichment. The AREB cis‐acting elements present in the promoters of drought‐inducible genes are recognized by *bZIP*‐type transcription factors, which include four AREB/ABF family members and the closely related ABI5 protein [116] (Figure 3b). During ABA signal transduction, SnRK2 protein kinases directly phosphorylate and activate AREB/ABF and ABI5, converting drought perception signals into transcriptional activation of the flavonoid biosynthetic pathway. Recent studies have further revealed that the highly conserved MYB‐bHLH‐WD40 (MBW) protein complex plays an indispensable role within this regulatory network [153]. The drought‐activated ABA signaling pathway closely intersects with the MBW complex, typically through upregulating the expression of complex‐specific MYB or bHLH subunits [154], thereby establishing a core transcriptional node that potently activates downstream late‐stage flavonoid biosynthetic genes. This regulatory framework is supported by evidence from potato (*Solanum tuberosum* L.), where grafting onto drought tolerant rootstocks, combined with metabolomic and transcriptomic profiling, revealed significant flavonoid accumulation in both leaves and roots of heterografted plants, alongside enhanced scion drought tolerance [155]. Weighted gene coexpression network analysis further identified *MYB* and *MYB*related genes as hub regulators of this droughtresponsive module, reinforcing the central role of the MYBWD40bHLH transcription factor complex in orchestrating drought stress responses through the flavonoid pathway [155].

Subcellular transport and compartmentalization are essential prerequisites for flavonoids to effectively fulfill their functions under drought stress. Flavonoids are biosynthesized on multienzyme complexes assembled on the cytoplasmic face of the endoplasmic reticulum and are subsequently translocated to their sites of action via membrane transporters, vesicular trafficking, and glutathione S‐transferases (GSTs) [156]; this precise spatial compartmentalization underlies the maintenance of their antioxidant activity [157]. Under prolonged drought, the expression of flavonoid biosynthetic genes exhibits phased dynamic changes, leading to gradual flavonoid accumulation. Such metabolic plasticity enables plants to cope with sustained drought stress and confers the potential to withstand multiple concurrent stresses [158, 159]. The functional relevance of these molecular mechanisms has been validated across diverse crop species. Under drought stress, Tibetan hulless barley (*Hordeum vulgare* L.) reconfigures phenylpropanoid metabolic flux by repressing lignin biosynthesis while simultaneously upregulating anthocyanin and other flavonoid pathways, thereby enhancing drought tolerance [160]. Similarly, in Arabidopsis, the strongly drought‐inducible gene *CsCYP75B1* activates flavonoid biosynthesis and augments overall antioxidant capacity [161]. Collectively, the MBW complex constitutes a central relay node bridging ABA‐mediated drought signaling and transcriptional activation of flavonoid structural genes: it functions downstream of AREB/ABF and SnRK2, directly engaging the terminal catalytic steps of flavonoid biosynthesis; concurrently, this node is subject to precision check‐and‐balance by both positive and negative regulatory inputs, enabling dynamic modulation of the drought response.

### 3.2 Flavonoids in Plant–Microbiome Interactions

Flavonoids serve multiple functions in plant–microbiome interactions. Beyond enhancing drought tolerance, they contribute to defense against bacterial and fungal pathogens [162] and act as signaling molecules that promote symbiotic associations with rhizobia, *Frankia* spp., and AMF, thereby improving host nutrient acquisition [134, 163]. AMF colonization in turn activates plant defense‐ and metabolism‐related genes, including flavonoid pathway enzymes such as *PAL* and *CHS* [164], while suppressing JA/ET signaling to attenuate host immune responses and facilitate fungal establishment [165, 166, 167]. Flavonoid accumulation also modulates endogenous levels of JA, SA, and ethylene, which play critical roles in plant defense and stress responses.

Genotype‑specific flavonoid exudation governs the selective recruitment of beneficial rhizosphere microbes within crop species [168]. Distinct genotypes produce divergent root flavonoid profiles in terms of compound composition and secretion abundance, enabling nutrient‑efficient varieties to selectively enrich phosphate‑solubilizing and nitrogen‑fixing microbiota [169]. Nitrogen‑efficient rapeseed genotypes secrete abundant kaempferol to specifically enrich *Bacillaceae* strains for improved nitrogen uptake, whereas flavonol‑deficient fls1 mutants fail to assemble this beneficial community, confirming flavonoids as the key metabolites mediating genotype‑dependent microbiome filtering [170]. Different maize (*Zea mays* L.) inbred lines exhibit over threefold variation in root flavonoid exudation; high‑flavonoid lines accumulate significantly more *Oxalobacteraceae* and Pseudomonas in the rhizosphere, facilitating root growth and nutrient mobilization under low‑nitrogen and drought conditions [171]. Tetraploid rice (*Oryza sativa* L.) upregulates root flavonoid biosynthesis relative to diploids, selectively recruiting rhizosheath promoting Pseudomonas to strengthen water and P capture under water deficit [172]. These observations suggest that flavonoid‑mediated rhizosphere recruitment is genotype‑dependent across crop species, yet the underlying mechanisms likely involve species‑specific regulatory networks. At the molecular level, plant growth‑promoting rhizobacteria (PGPR) sense and chemotactically respond to flavonoid gradients in root exudates via dedicated receptors (e.g., NodD in *Rhizobium*, Y4lC in *Bacillus*), activating symbiotic gene cascades for targeted colonization [173], mycorrhiza helper bacteria, including *Streptomyces*, *Burkholderia*, and *Paenibacillus* spp., facilitate AMF and ectomycorrhizal fungi establishment through spatiotemporally coordinated signaling [174]. Leguminous plants secrete specific flavonoids as host recognition cues for symbiont selection in nitrogen‑fixing rhizobia [175], indicating a divergence between legume and non‑legume crops in this mechanism. Under nitrogen limitation, plants coordinate lateral root development and flavonoid secretion via the *LRT1* locus, concomitantly enriching flavonoid‑dependent *Oxalobacteraceae* [171]; given that drought frequently coincides with reduced soil nitrogen availability and rhizosphere microbiome dysbiosis, this regulatory axis may represent an adaptive strategy for combined water‑nutrient stress. A thorough understanding of such plant–microbe interaction mechanisms may provide a theoretical foundation for developing drought tolerant and growth promoting microbial inoculants [176].

## 4 Challenges in Arid Areas: Soil Degradation

**FIGURE 4:**
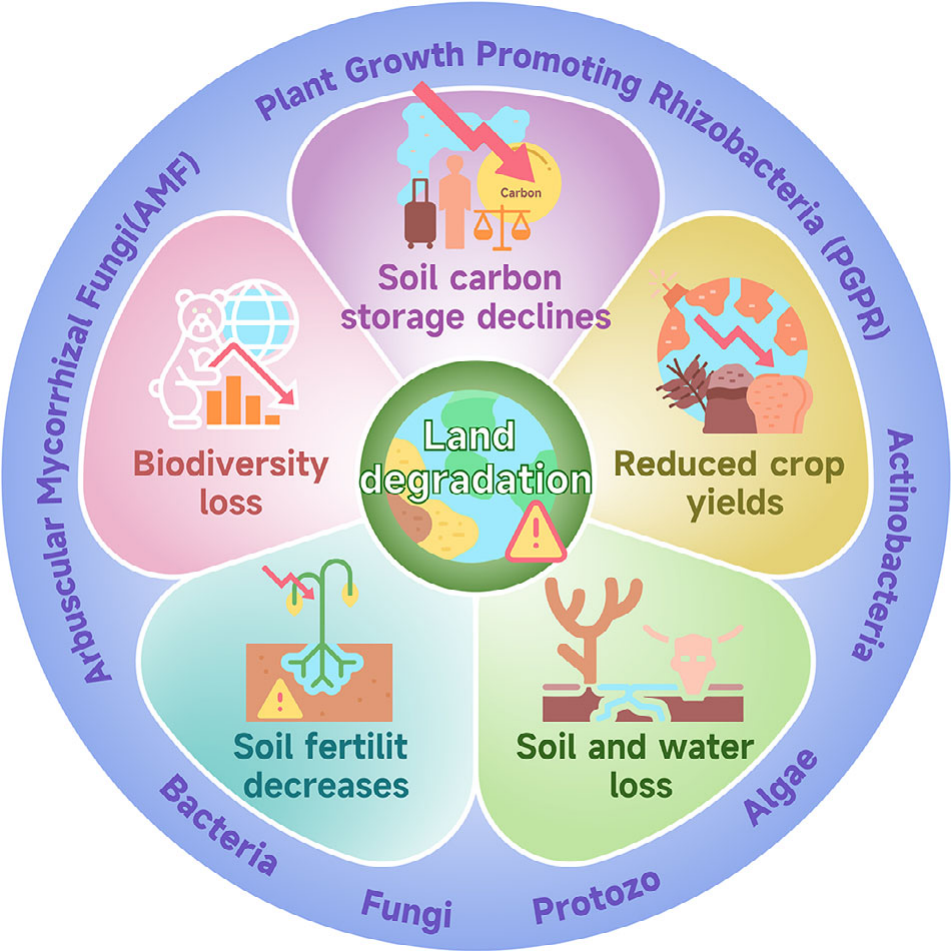
Principal types of land degradation (inner ring) and primary types of plant beneficial microbiota (outer ring).

Healthy soil serves as the cornerstone of ecosystems, supporting plant communities, and soil microbial activity while maintaining critical environmental functions such as water purification, carbon sequestration, and nutrient cycling [177, 178, 179]. Soil microbial community is an indicator of ecosystem health and recovery as soil microbiome can regulate soil nutrient cycling, decompose organic matter, inhibit soil borne plant diseases, change soil structure, and promote plant biomass growth [180]. However, agricultural land use, industrial production, and urbanization have brought serious threats to soil health, resulting in surface water pollution, reduced plant growth or increased plant mortality, changes in soil microbiome communities and carbon cycling, lowering biodiversity, thus affecting entire ecosystems [181]. Soil degradation refers to the decline of soil productivity or land use, or even the complete loss of soil's physical, chemical and/or biological activities [182]. Degradation is a dynamic and complex process and include various processes reducing soil quality, such as soil erosion, salinization, acidification, desertification and nutrient deficiency [183] (Figure 4). The total area of global soil degradation currently covers one third of the Earth's land surface, seriously affecting the soil's water storage and filtering functions, especially in ecologically fragile areas [184].

In soil restauration process of forests and farmlands, the interactions between the soil biological communities and soil hydrological functions plays an important role [185], and thus understanding the dynamic process of the interaction between soil microorganisms and plant rhizosphere lays a foundation for the restoration of soil ecosystems [178, 181]. Root exudates play a pivotal role in carbon allocation and soil amelioration, as plants release up to 20% of their photosynthetically fixed carbon into the rhizosphere through the exudation of organic compounds [186]. These exudates not only promote soil aggregate formation and enhance water retention capacity but also selectively recruit drought‐tolerant functional microbes, establishing a “root–microbe–soil” synergistic network for drought resistance (Figure 4). The abundance and community composition of microorganisms directly determines soil activity and fertility, ultimately influencing plant survival, growth, and inherent defense capabilities. Among these, the rhizosphere microbiota plays a particularly prominent role, participating in multiple beneficial physiological processes including nitrogen fixation, P solubilization, and phytohormone synthesis, thereby enhancing plant stress tolerance [187]. One study used Illumina MiSeq high‐throughput sequencing to characterize rhizosphere microbial communities and their root interactions in sugarcane (Saccharum officinarum L.) across different drought stress gradients [188]. Sequencing results indicated that moderate drought (soil moisture maintained at 50% of field capacity) reduced rhizosphere bacterial Chao1 and Shannon indices by 22.4% and 16.7%, respectively, relative to well‐watered control conditions. Taxonomic annotation showed that the relative abundances of drought‐tolerant taxa, including *Streptomycetales*, *Sphingomonadales*, and *Rhizobiales*, increased by 3.1‐fold, 2.6‐fold, and 2.2‐fold, respectively, establishing them as signature indicator taxa in the drought‐stressed rhizosphere. Furthermore, inoculation with *Sphingomonas* Hbc‐6, a strain isolated from a desert plant, reversed the drought‐induced attenuation of rhizosphere microbial diversity. Quantitative high‐throughput sequencing confirmed that this bacterium increased biomass under both normal and drought conditions; compared with the control group, under drought stress, this strain increased root fresh weight by 61.3% and shoot dry weight by 96.3% [189]. Rhizosphere microorganisms also activate genes associated with dormancy and osmotic regulation, optimizing nutrient allocation and exudation patterns within plants [190, 191], thereby systematically enhancing tolerance to external stresses such as drought. This cascade of responses forms an integrated network extending from soil functional restoration to improved plant stress resilience. Notably, the assembly and function of such rhizosphere microbial ecological networks are dynamically modulated by a combination of biological and environmental factors.

Abiotic and biotic factors such as soil type and cultivated plant varieties affect nutrient availability in the rhizosphere soil, such as the contents of available N, P, potassium (K) and organic C [192]. Soil moisture, air and nutrients in turn play an important role in plant rhizosphere microbial community [193, 194], plant growth and secondary metabolite release, and thus choosing suitable soil amendments (Section 3.1) and irrigation techniques (Section 3.2) should be considered in order to improve drought tolerance. As understanding the dynamic processes of soil microbial interactions with plant rhizospheres lays the foundation for the remediation of soil ecosystems [195], it would be important to understand how soil remediation in arid areas could be improved by modulating microbial‐plant rhizosphere interactions [196, 197].

## 5 Measures to Alleviate Drought Stress in Arid Areas

This section focuses on practical strategies to alleviate drought stress, encompassing the mechanisms by which soil amendments enhance plant nutrition and drought resistance, the optimization of water‐saving irrigation techniques, and the principles for screening drought‐tolerant plants. Therefore, careful selection of the right type of soil amendments, irrigation methods, and cultivars can help in improving drought tolerance, which we discuss in more detail in the sections below.

### 5.1 Soil Amendments

Common organic amendments include compost, green manure, earthworm dung, peat soil, and biochar among others (Table 2). Organic amendments in soil can adsorb pollutants such as heavy metals (e.g., lead, cadmium) [198, 199], organic contaminants (e.g., polycyclic aromatic hydrocarbons, pesticides), and other harmful compounds [200, 201], thereby fixing them in the soil and preventing groundwater pollution. They improve soil structure and fertility by promoting the formation of soil agglomerates [202], enhance cation exchange capacity, and optimize pore structure and water‐holding capacity [203, 204], thereby systematically improving soil productivity and plant performance [205, 206]. Accordingly, organic amendments can be used for drought prevention and management.

**TABLE 2:** Common organic soil amendments.

Organic Ameliorants	Source	Function	pH Range	Common nutritional values			Applicable soil types
				N(%)	P(%)	K(%)	
Compost	Organic materials	Reduce heavy metal content	6.5–8.0	2.00	0.5–1.00	2.00	Various types
Green‐manure	Green‐manure crops	Supplement nutrients	6.0–7.5	2.75	0.60	1.91	Various types
Vermicompost	Earthworms	Promote microbial growth	5.5–7.5	1.00–2.00	0.30–0.80	0.50–1.20	Various types
Peat	Plant residues	Preserve moisture and fertility	3.5–5.5	0.30–1.00	0.05–0.20	0.10–0.50	Sandy soils, infertile soils and Cohesive soil
Biochar	Organic materials	Enhance carbon sequestration	7.5	ND	ND	ND	Acidic soils, heavy metal contaminated soils

*Note*: ND, no data

In arid regions, biochar, compost, and green manure are recommended soil amendments due to their ability to comprehensively improve soil physical, chemical, and biological properties. Biochar enhances soil water retention, reduces evaporation, and increases fertility, concomitantly enriching drought‐tolerant bacterial taxa (Actinobacteria, Firmicutes) and functional diversity [207]. Compost and legume green manure improve soil properties for sorghum (*Sorghum bicolor* L.), increasing nitrogen uptake and water use efficiency while promoting fungal–bacterial networks. Vermicompost improves aeration, water retention, and yield quality in cucumber (*Cucumis sativus* L.) [208], increasing microbial richness.

Soil amendment efficacy hinges on matching their structural‐chemical properties to the target soil constraint (e.g., carbon framework, aliphatic/aromatic configuration). For example, humus (increasing cation exchange capacity to 20–40 cmol/kg) or biochar (regulating pH fluctuation ≤± 0.5) are preferentially used in salinized soils due to their good ability to increase water retention capacity [209] by optimizing the pore structure, and ability to improve nitrogen and P mineralization and trace element (e,g. Fe and Mn) release [210, 211, 212]. Addition of these amendments also enriches salt‐tolerant flora (e.g., *Halomonas*) and promotes proline synthesis (↑two to three times), which in turn enhances plant osmoregulation [212]. In cauliflower (*Brassica oleracea var. botrytis*) grown in calcareous soil, exogenous application of humic acid and nanocalcium significantly promotes growth and yield, with an increase of approximately 30% [213].

Alginate represents another category of organic amendments. It is an anionic polysaccharide extracted from brown algae, rich in organic components and plant hormones. This amendment promotes the formation of soil aggregate structures, improves soil aeration and water permeability [214], and enhances soil water retention through its high hygroscopicity and gel‐forming capacity, thereby helping to maintain soil moisture during drought periods. For instance, a novel bio‐based hydrogel has been fabricated with sodium alginate and alkali lignin as the main structural matrix [215]. Tests show that this composite hydrogel exhibits superior water absorption performance compared with conventional sodium alginate hydrogels. It effectively improves soil water retention, raises seed germination rates by 19.04%, mitigates soil drought stress, and facilitates crop growth [215]. However, despite the promising drought‐resistance amelioration effects exhibited by alginate‐based hydrogels under controlled laboratory conditions, their field‐scale application remains constrained by economic viability, agronomic compatibility, and ecological risks [216]. Specifically, the nutrient release kinetics of these materials are poorly matched with crop demand patterns, and their water retention efficacy is readily compromised by field soil heterogeneity, indigenous microbial activity, and climatic fluctuations, resulting in insufficient functional stability [217]. Concurrently, the degradation of these materials poses dose‐ and time‐dependent ecological risks, including soil microecological imbalance, impeded nutrient cycling, reduced soil porosity, inhibited root development, and heavy metal mobilization and contamination, which collectively impose severe constraints on their large‐scale field deployment [218]. In contrast, humic substances possess a hydrophobic–hydrophilic composite structure that not only drives the formation of soil aggregates and the reconstruction of microbial metabolic networks but also increases soil water‐holding capacity through their sponge‐like porous structure and reduces water evaporation via surface film formation [219]. Furthermore, humic substances promote the accumulation of compatible solutes in plant tissues, maintaining cell turgor under water deficit conditions and thereby significantly improving drought resistance. This is supported by field evidence showing that humic acid application rapidly promotes macroaggregate formation [220], optimizes pore characteristics, reduces bulk density, and improves the hydraulic properties of sandy soils, ultimately leading to a 28%–54% increase in wheat (*Triticum aestivum* L.) yield.

### 5.2 Organic Amendments Alleviate Drought Stress Via Enhancement of Flavonoids Accumulation

Organic amendments enhance plant antioxidant capacity and water retention by promoting endogenous flavonoid synthesis, thereby mitigating the adverse effects of drought stress on growth and development. For example, humic acid, chitosan, and melatonin, each representing distinct categories of amendments, have been shown to promote flavonoid accumulation through different mechanisms.

Humic acid acts as a soil conditioner that primarily promotes flavonoid accumulation through transcriptional reprogramming of root secondary metabolism [221, 222, 223]. Transcriptomic profiling of tomato (*Solanum lycopersicum* L.) roots demonstrated that humic acid treatment significantly modulated carbohydrate metabolism and secondary metabolism—particularly the phenylpropanoid and flavonoid pathways—within 24 h of application [224]. At the enzymatic level, sodium humate regulates the activities of key enzymes in the phenylalanine pathway, including HCT (hydroxycinnamoyltransferase) and F5H (ferulate‐5‐hydroxylase), thereby influencing flavonoid biosynthesis [224]. Furthermore, humic substances have been shown to enhance both the activity and gene expression of *PAL*, a ratelimiting enzyme in the phenylpropanoid pathway, resulting in elevated concentrations of phenolics and their amino acid precursors [225]. Collectively, these molecular changes redirect root metabolic flux toward flavonoid production.

As a typical biotic elicitor, chitosan is first perceived by receptors on the plasma membrane plant. This recognition step activates Ca^2^^+^ channels, plasma membrane NADPH oxidase and cytosolic nitrate reductase, triggering a rapid burst of secondary messengers including Ca^2^^+^, H_2_O_2_ and NO [226]. These signaling molecules further initiate upstream MAPK cascades, which upregulate the expression of core structural genes *PAL*, *CHS* and *F3H* in the phenylpropanoid pathway [224]. Meanwhile, the accelerated tricarboxylic acid (TCA) cycle provides abundant energy and carbon skeletons to sustain secondary metabolism. Coordinated transcriptional reprogramming and central carbon redistribution jointly elevate the metabolic flux from phenylalanine to flavonoids, leading to remarkable accumulation of flavonoids and anthocyanins.

Distinct from humic acid and chitosan, melatonin serves as a direct signaling molecule that modulates flavonoid biosynthesis via receptor‐dependent transcriptional activation. Transcriptomic profiling of wheat (*Triticum aestivum* L) revealed that exogenous melatonin treatment triggers the activation of tryptophan metabolism and flavonoid synthetic pathways [227]. Co‐expression network analysis further demonstrated that multiple transcription factor families, such as *AP2/ERF*, *WRKY*, *bZIP*, *C2H2*, *bHLH*, *NAC* and *MYB*, function cooperatively to orchestrate the expression of differentially expressed genes. Integrated transcriptome and metabolome assays conducted on maize (*Zea mays* L.) roots delivered consistent supporting results [228]. Melatonin markedly upregulated a full set of core genes governing flavonoid production, including *PAL*, *C4H*, *4CL*, *HCT*, *CHS*, *CHI*, *F3′5′H* and *DFR*. Meanwhile, it stimulated drought‐responsive transcription factors (*ERFs*, *NACs*, *MYBs*, *bHLHs*) and reshaped the transcriptional abundance of genes participating in phytohormone signal transduction. Supporting this, an ajwain (*Carum copticum* L.), combined applications of melatonin, chitosan, humic acid, and selenium were found to stimulate flavonoid synthesis, enhance antioxidant capacity, and significantly improve physiological traits and seed yield under drought conditions [229]. By regulating osmotic pressure, promoting cell expansion, and boosting antioxidant enzyme activity, these amendments collectively help sustain normal physiological functions in plants under water deficit.

### 5.3 Organic Amendments Enhance Soil Microbiome

Another way organic amendments may improve plant nutrition and thus tolerance against drought and other abiotic and biotic stresses is via improvement of the soil microbiome. Organic amendments not only directly enrich specific microbial communities but also indirectly influence bacterial composition by altering soil properties [230]. These changes are closely related to multiple soil functions. One study confirmed that organic amendments enrich the key genus *Lysobacter* and alter soil carbon and nitrogen cycling processes [231], increasing bacterial α‐diversity, improving plant growth, suppressing pathogens, optimizing soil quality and microbial diversity, and ultimately enhancing overall ecosystem multifunctionality. A key function of these beneficial microbes, particularly PGPR, including phosphate solubilizing bacteria (PSB) and potassium‐solubilizing rhizosphere bacteria (KSR), is the conversion of soil nutrients into plant‐available forms. For example, although P is an essential macronutrient for plants [232], only a small fraction is bioavailable because most P is fixed in insoluble complexes. PGPR can solubilize insoluble phosphate complexes into soluble forms [233] by producing organic acids that chelate mineral ions and lower the pH of the medium [234], or by mineralizing organic P via phosphatase or phytase secreted by soil microorganisms [235, 236]. Similarly, KSR—which are more abundant in rhizosphere soil than in bulk soil [237], can decompose aluminosilicate minerals, releasing soluble K from insoluble forms [238]. The production of organic acids such as citrate, oxalate, and acetate by soil microbes, including certain *Bacillus* spp., helps convert insoluble K into soluble, plant‐available K, thereby improving soil fertility and crop productivity [239].

Besides macronutrients such as P and K, microbes also aid the acquisition of plant micronutrients. For instance, zinc (Zn) is an essential micronutrient, participating mainly in carbohydrate metabolism, cytochrome synthesis, and detoxification of superoxide radicals, while also serving as a coenzyme for many enzymes, and promotes the secretion of growth hormones among other functions. Zn solubilizing rhizobacteria (ZSR) can alleviate Zn deficiency symptoms and increase plant biomass [234, 240] and dissolution of Zn can be achieved through various mechanisms such as secretion of metabolites, proton efflux, or production of chelating compounds, leading to the breakdown of Zn complexes [241]. In addition, the production of organic and inorganic acids such as sulfuric acid, carbonic acid, and nitric acid by ZSR also promotes the dissolution process of Zn. For instance, siderophores have been shown to participate in chelating Zn compounds, thereby enhancing plant Zn elements [242]. Perhaps the best studied plant beneficial microbes are root‐colonizing mycorrhizal fungi, which play important part also in plant's ability to adapt to drought conditions. “Mycorrhiza” was first proposed by German botanist and biologist Albert Bernhard Frank in 1885 [243]. Based on phylogeny and host interactions, these fungi are classified into five categories: AMF, Ectomycorrhizae, Ericoid mycorrhizae, Orchid mycorrhizae, and Arbutoid mycorrhizae [244, 245].Among these, AMF are the most widespread and ecologically relevant for agricultural plants, forming symbiotic associations with the majority of crop species, including wheat (*Triticum aestivum* L.), maize (*Zea mays* L.), rice (*Oryza sativa* L.). Mycorrhizal fungi colonize host root systems, and multiple mycorrhizal types coexist in many ecosystems. Some plants can form symbiotic relationships with more than one type of mycorrhiza. A single fungus can connect different underground plants, forming a common mycorrhizal network through which nutrients are exchanged [246]. Plants enhance their uptake of key nutrients like P and nitrogen through mycorrhizal symbiosis, which is essential for adapting to drought conditions. Mycorrhizal fungi enhance the P acquisition of the host plants by secreting phosphatases, which release inorganic P from organic sources such as phospholipids, nucleic acids, and proteins. These fungi also collaborate with PSB to convert P into a form that can be absorbed by the fungi and transported to the host plant [247].

Among mycorrhizal fungi, AMF and EMF are the two most widely reported mycorrhizal types. EMF mainly associate with woody perennials and forest trees, including walnut, chestnut, hazelnut, oak, pine, and poplar, participating in organic matter decomposition and nitrogen cycling while improving drought tolerance [197, 248]. In turn, the vast majority of agricultural plants are associated with AMF, which has been shown to improve drought tolerance, for example, in major cereal and legume crops including maize (*Zea mays* L.), wheat (*Triticum aestivum* L.), and soybean (*Glycine max*). These fungi can establish mutualistic relationships with the roots of most plant species. Therefore, soil amendments suitable for improving AMF colonization such as low‐dose biochar [249], well‐matured composts rich in humic substances [250], or rock‐phosphate–enriched organo‐mineral formulations that simultaneously supply slow‐release P and maintain neutral to slightly alkaline pH [251], would be recommended in arid areas. AMF can improve water retention capability of the soil by producing a substance called glomalin‐related soil protein, which acts as “glue” in the soil by physically wrapping mycelium, promoting the formation of water‐stable agglomerates in the soil, enhancing water retention capacity, stabilizing soil structure, and improving soil properties [252, 253]. The colonization of AMF in the root system can change PSMs and promote the production of flavonoids [254, 255]. Moreover, AMF also has the ability to improve plant antioxidant activity, promote the accumulation of osmotic regulatory substances and regulate the expression of drought stress‐related genes such as *GintAQPF1*, *GintAQPF2*, *TdDRF1*, and *MdGH3‐2/12*, which help to maintain the water balance and photosynthesis efficiency under drought stress [256]. It can be said that AMF are “drought‐resistant bodyguards” for plants, significantly improving drought tolerance. For example, inoculation with *Funneliformis mosseae* was shown to induce flavonoid synthesis in trifoliate orange (*Poncirus trifoliata* L.), a citrus rootstock, under water deficit [165]. This response involves activation of the phenylpropanoid pathway, enhancing the activities of *PAL*, *C4H*, *4CL*, *CHI*, and leads to flavonoid accumulation, which scavenges reactive oxygen species and alleviates oxidative damage. Consequently, this maintains water balance and photosynthetic efficiency, contributing to markedly improved drought tolerance. Similarly, AMF colonization significantly altered root secondary metabolite profiles in olive (*Olea europaea* L.), increasing flavonoid and total phenolic content, scavenging ROS, and enhancing cellular water retention and antioxidant capacity, thereby enabling olive trees to perform well under semi‑arid Mediterranean conditions [255].

If plant residues rich in flavonoid metabolites (such as compost and green manure) are composted and applied to the soil, this will not only increase organic matter, but also release flavonoids [257], which in turn may positively affect the structure of soil microbiome communities and their functional structure, and thus lead to nutrient transformation [258, 259, 260]. The latter effect may raise from the ability of the microorganisms to convert elements that insoluble but necessary for plant growth, such as P, K, and Zn, into a form that plants absorption [261]. Therefore, flavonoid metabolites may affect the absorption and utilization of nutrients, which in turn may enhance plant resistance to stressors, such as drought. Flavonoid metabolites play a significant role in soil improvement by promoting the formation of biofilms of nitrogen‐fixing bacteria. Biofilms of these bacteria protects the bacterial nitrogenase enzyme, which facilitates the interactions between plant roots and nitrogen‐fixing bacteria by affecting bacterial root colonization, thereby achieving effective biological nitrogen fixation [254, 206]. Under drought stress, this biofilm‐mediated protection becomes particularly critical, as water deficit severely inhibits nitrogenase activity and reduces nitrogen availability. By sustaining nitrogen fixation, flavonoids indirectly support the synthesis of osmotic regulators (e.g., proline) and stress‐responsive proteins, thereby contributing to drought tolerance. For example, in rice (*Oryza sativa* L.), rootsecreted flavonoids such as apigenin induce bacterial biofilm formation and promote rhizosphere colonization. This process alters root microbiome structure and increases grain yield under nitrogenlimited conditions [263]. Flavonoids can also act as signaling molecules to recruit specific microbial groups, thereby improving the soil microbial community and plant nutrient absorption. For example, genistein and quercetin can significantly affect microbial diversity in the soil, increasing the abundance of beneficial microorganisms [254], whereas application of apigenin and luteolin can increase the abundance of *Oxalobacteraceae* in the maize (*Zea mays* L.) rhizosphere, promoting plant nutrient absorption and growth [171]. Furthermore, flavonoids may also interact with organic matter and minerals in the soil and improve the soil's physical and chemical properties. They increase soil aggregation, enrich the content of organic matter and total nitrogen in the soil, and increase available nutrients, thereby enhancing soil fertility and water retention capacity [264].

Combining microbial inoculants with humic substances can enhance the biological activity, cell proliferation, and differentiation of plant roots, stimulate and promote plant growth, and increase yield [265]. Under drought stress, a well‐developed root system improves water uptake from deeper soil layers. Zhao and Naeth (2022) [266] studied the effects of adding nano humus on the growth of alfalfa (*Medicago ruthenica* L.) and barley (*Hordeum vulgare* L.), and found that combining AMF with nano humus can increase plant biomass and alter soil physicochemical properties by significantly increasing soil cation exchange capacity, total organic carbon, as well as N, P, k contents. These improvements enhance soil water‐holding capacity, which is critical for maintaining plant hydration under drought conditions. The combination of AMF and nano humus have also been shown to increase root and stem biomass on alfalfa (*Medicago sativa* L.), and barley (*Hordeum vulgare* L.), latter species also experiencing a substantial increase in seed yield. A larger root system, in turn, allows plants to access water from greater soil depths during dry periods, hence increasing drought tolerance.

### 5.4 Irrigation Technology

Appropriate irrigation regimes can be widely adopted to mitigate drought stress, while simultaneously boosting intrinsic plant drought resistance by stimulating flavonoid biosynthesis. This regulatory relationship is supported by existing evidence confirming that drought stress reshapes the accumulation of phenolic compounds including flavonoids [267, 268, 269]. For example, on jujuba (*Ziziphus jujuba* Mill.), it has been shown that the levels of epicatechin, catechin, rutin, quercetin, and cinnamic acid vary depending on the irrigation method used [270]. Kapur [271] further showed that the total soluble solids, carbohydrates, flavonoids and other contents of strawberry (*Fragaria × ananassa* (Weston) Duchesne ex Rozier) were significantly increased by different drip irrigation levels and biostimulation (seaweed extracts), while the taste of strawberries and the content of health‐related compounds (e.g., antioxidant activity, phenols) were significantly increased.

At present, magnetized irrigation [272] and nanobubble irrigation [273] are two relatively advanced irrigation technologies used in agriculture. Magnetized irrigation is an innovative technology that improves agricultural efficiency through physical and biochemical modification of water. This irrigation technique reduces the size of water molecules and changes their structure and physical properties, enhancing the interaction between water molecules and ions [274]. This structural change reduces the viscosity of water, increases water permeability and solubility [275], penetrates soil pores more effectively, promotes salt leaching (e.g., Na^+^, Cl^−^), and alleviates soil salinization [272]. At the same time, enhanced water mobility disrupts soil aggregates, increases porosity, and improves rhizosphere air‐water exchange [276]. The improved soil structure creates a more aerobic microenvironment that selectively enriches beneficial aerobic microorganisms (e.g., *Pseudomonas, Bacillus*) while inhibiting anaerobic pathogens (e.g., *Fusarium, Rhizorotinia*) [277]. In addition, magnetized water can directly inhibit the viability of pathogens by altering the permeability of microbial membranes or soil enzyme activities [278]. Combined with microbial organic fertilizer, magnetized irrigation can enhance the activity of PGPR. These PGPR may secrete antagonistic compounds (e.g., antibiotics, siderophores) to exclude pathogens and stimulate systemic resistance in plants, further enhancing flavonoid biosynthesis [279]. The cumulative effect of magnetized water improved soil porosity, optimized microbial balance and nutrient mobilization, and jointly improved root uptake efficiency. This provides an additional carbon skeleton and energy for the synthesis of secondary metabolites such as flavonoids, as evidenced by the increased flavonoid content in magnetized water‐treated jujube fruits [276].

Nanobubble water irrigation is a new irrigation technology that uses micro‐nanobubble generation devices to inflate irrigation water [279]. Nanobubbles are a type of bubbles with a diameter of less than 1 micrometer, and can quickly achieve supersaturation of dissolved oxygen and possess electrical, oxidative, and bactericidal properties. They have high stability in water [280], and can enhance gas solubility [281], and generate ROS [282], but the concentration of ROS is typically within a range that is not harmful to plant roots but rather beneficial for promoting various physiological processes [283], enhancing plant metabolic activities (e.g., promotes synthesis of flavonoid metabolites), seed germination and growth [284]. Nanobubble irrigation also improves the utilization rate of organic fertilizers by soil microorganisms [275]. Oxygen nano‐spark water irrigation has also been shown to change the redox conditions of the soil, increasing the oxygen content in the soil, and reduce methane emissions, as shown in flooded shallow rice (*Oryza sativa* L.) fields [283].

Plants treated with hydrogen water may exhibit higher antioxidant activity and lower lipid peroxidation levels [285]. Hydrogen selectively scavenges ROS through its reducing activity, activates antioxidant enzymes, and up‐regulates the expression of genes related to glutathione (GSH) metabolism, thereby enhancing the plant's tolerance to drought and other abiotic stress [279, 286]. This technology shows potential in particularly arid regions as it can reduce water usage by about 30% while improving soil aeration and salt alkali tolerance [287]. In tomato (*Solanum lycopersicum* L.) [273], a comparative study of freshwater, oxygen nanobubble, and hydrogen nanobubble irrigation identified 2132 differentially expressed genes (DEGs) between oxygen and hydrogennanobubble treatments; among these, 1016 DEGs were specifically enriched under hydrogen nanobubble treatment, which was associated with enhanced antioxidant capacity and improved yield [273]. Notably, hydrogen nanobubble irrigation upregulated the *CYP73A* gene, leading to reduced lignification and increased flavonoid production. Therefore, hydrogen nanobubble irrigation may have more beneficial impacts on agricultural plants compared to oxygen nanobubble method under drought conditions, warranting further testing. However, these irrigation technologies remain at the experimental‐pilot stage, with empirical evidence predominantly derived from controlled environments. Their widespread adoption is constrained by high initial costs, absence of standardized protocols, and insufficient large‐scale field validation, warranting comprehensive cost‐benefit analyses for specific agricultural systems.

### 5.5 Integrating Flavonoid Metabolism and Microbiome Engineering for Drought‐Resilient Crops

Drought stress induces dynamic shifts in plant resource allocation between immediate and long‐term survival. For example, under water deficit conditions, plants such as *Arabidopsis* [288] and maize (*Zea mays* L.) [289] preferentially allocate photoassimilates (e.g., soluble sugars) to root growth rather than leaf expansion, thereby increasing the root‐to‐shoot biomass ratio. While this reallocation enhances water foraging capacity, it reduces carbon investment in leaf‐based secondary defense compounds (e.g., phenolics, alkaloids) [290]. Consequently, drought‐adapted plants may face trade‐offs between drought and biotic stresses: in wheat (*Triticum aestivum* L.), prioritization of root biomass correlates with a 30% decrease in leaf JA levels, weakening resistance to aphids (*Schizaphis graminum*) [291, 292]. These morphological and metabolic adjustments may cascade to ecosystem‐level impacts [293]. The shift toward root‐derived exudates (e.g., amino acids during drought recovery) alters soil nitrogen cycling, favoring microbial taxa capable of metabolizing simple C/N substrates (e.g., *Pseudomonas* over *mycorrhizae*) [294]. Meanwhile, reduced litter input and altered root exudate profiles can lead to decreased soil organic carbon sequestration in drought‐affected agroecosystems [295]. During drought, root exudates undergo significant metabolic reprogramming, reflecting the trade‐off between defense and nutrient acquisition. In the early stages of a drought, plants reduce the secretion of amino acids and organic acids, reallocating carbon to secondary metabolites and osmoprotectants (such as soluble sugars and polyamines [296]). For example, a recent study on soybean (*Glycine max*) found that high rhizosphere microbial diversity triggers the exudation of 2‐naphthoic acid under drought conditions, which selectively recruits the beneficial bacterium *Sinorhizobium* CS204 [297]. This recruitment enhances nitrogen fixation and nutrient acquisition, significantly improving soybean (*Glycine max*) drought tolerance.

Drought stress also increases synthesis of flavonoids, which mitigate oxidative damage and regulate rhizosphere signaling [298]. These drought‐induced changes in root exudates further alter the structure of soil microbial communities [299]. Flavonoids also enhance recruitment of plant beneficial AMF through quercetin signaling [300, 301, 302] as well as by remodeling root architecture, providing a dual selection criterion for drought‐tolerant crops [34]. Targeted cultivation strategies—such as grafting drought‐susceptible varieties into drought resistant rootstocks. For example, crafting tomatoes on drought resistant *Solanum torvum* rootstocks onto have been shown to significantly increase root flavonoid levels [303], demonstrating the flavonoid traits available in specific genotypes to adapt to drought‐affected agroecosystems. Similar success has been achieved in other solanaceous crops [304], when commercial cultivar ‘Ajay’ was grafted onto wild rootstocks of *Solanum macrocarpon*, *S. gilo*, and *S. torvum* and exposed to a 2‐year water deficit‐irrigation trial. The wild rootstocks significantly elevated root flavonoid and total phenolic contents, increased root biomass by 15%–30%, reduced fruit yield losses by 12%–44%, and improved water‐use efficiency by 6–10 kg m^−^^3^. Thus, grafting is an efficient tool for improving drought tolerance in agricultural systems. However, its current application is primarily limited to high value horticultural crops, particularly Solanaceae (e.g., tomato, eggplant, pepper) and Cucurbitaceae (e.g., cucumber, watermelon) [305]. Since the 1930s to 1960s, grafting has been commercialized in Asia (e.g., Korea, Japan) [306]. Grafting is also widely used in fruit trees to enhance drought tolerance. In citrus (*Citrus reticulata* Blanco), rootstocks such as ‘Carrizo’ citrange and ‘Sour Orange’ improve scion drought tolerance by inducing antioxidant activity and maintaining photosynthetic efficiency, showing tolerance to drought and other abiotic stresses [307, 308]. In contrast, grafting is rarely used in major field crops (wheat, maize, rice, soybean) due to their herbaceous growth habit, lack of compatible woody rootstocks, and high operational costs. Nevertheless, the principle of using rootstocks to enhance flavonoid‐mediated drought tolerance could potentially be extended to other crops through targeted rootstock breeding and improved grafting techniques. Future research should explore grafting in additional plant families and assess its techno‐economic feasibility under drought‐prone agricultural systems.

The current availability of ‘omics’ tools can enable higher precision in selection of drought related metabolomic or genetic indicators and could be used to a higher extent in generation and selection of drought resistant cultivars. For instance, metabolomics tools have allowed identification of cultivars with specific root flavonoids (e.g., quercetin‐3‐glucoside) associated with drought recovery (e.g., *Citrus × aurantium* Engl.) [309, 310], whereas metagenomics has been used to screen for plant genotypes (e.g., in *Triadica sebifera*) with higher release of flavonoid exudates and thus higher AMF colonization [311]. Moreover, *CRISPR/Cas9* multiplex gene editing technology could be used to a greater extend to target and edit key genes that increase flavonoid productions, as shown with soybean (*Glycine max*) where this tool was used to increase isoflavone content in leaves and seeds to enhance resistance to mosaic virus [312]. Utilization of multiple omics tools could help in generating an integrated approach that screens and utilizes key flavonoids (or other general stress metabolites) as biomarkers for selecting or engineering more drought resistant varieties, microbiome synergy, and resource efficiency [313].

## 6 Bioremediation Methods

**FIGURE 5:**
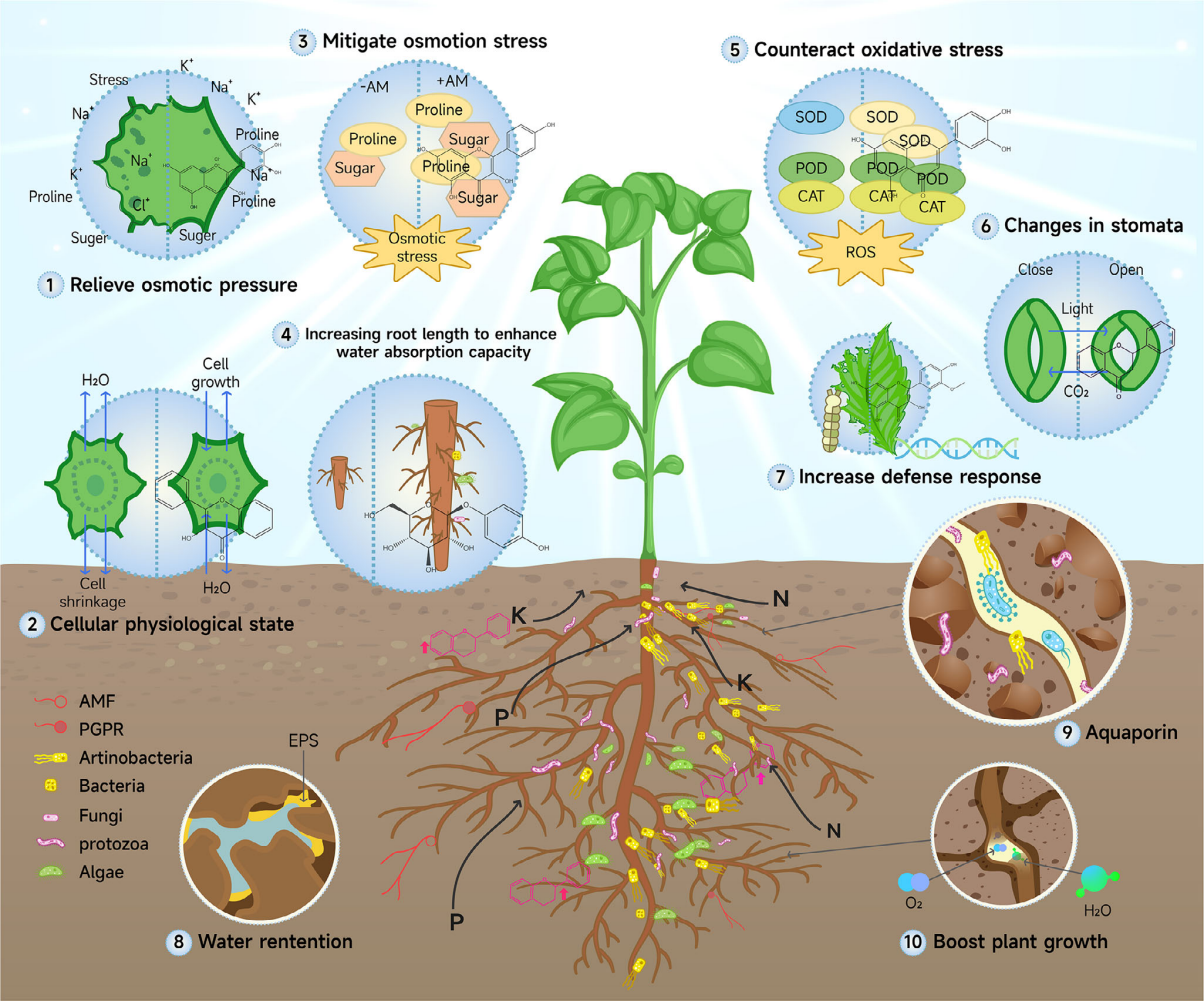
This figure distinguishes plant intrinsic drought responses (left) from microbe‐mediated enhancements (right). Vertical dashed lines indicate positive effects of microbial or flavonoid addition. Flavonoids act as recruitment signals, antioxidants, and regulators of root–microbe communication. ① Osmotic adjustment, plants accumulate proline, glycine, and sucrose, which cooperate with extracellular Ca^2^^+^ and water molecules to cope with osmotic changes; ② Microbe‐enhanced osmotic adjustment, AMF and PGPR induce additional accumulation of proline and soluble sugars beyond basal plant levels; ③ Microbe‐enhanced oxidative stress relief, AMF and PGPR increase antioxidant enzyme activities (SOD, POD, CAT) to combat ROS, and flavonoids (e.g., quercetin) contribute directly to ROS scavenging and stabilize these enzymes; ④ Microbe‐mediated cell water retention, AMF and PGPR help maintain cellular hydration under drought by improving root water uptake and inducing osmolyte accumulation; ⑤ Root morphological adaptation, under drought, plants increase root length and root hair density to enhance water uptake, which also promotes root exudation and microbial recruitment; ⑥ Microbe‐mediated defense enhancement, increased microbial diversity promotes upregulation of plant disease‐resistance genes, and flavonoids in root exudates act as signaling molecules to recruit beneficial microbes; ⑦ Stomatal regulation, stomata close during drought to reduce water loss. Microbe‐mediated responses; ⑧ Microbe‐mediated soil water retention, soil bacteria (e.g., Pseudomonas, Bacillus) secrete EPS that improve soil water retention, helping plants access water; ⑨ Microbe‐mediated aquaporin regulation, AMF and PGPR regulate plant aquaporins at gene expression, protein activity, and whole‐plant transport levels, improving water metabolism; ⑩ Microbe‐enhanced nutrient and water uptake, AMF form symbiotic associations with roots, extending hyphal networks to absorb more N, P, K, and water. Abbreviations: AM, arbuscular mycorrhizal, non‐colonized by arbuscular mycorrhizal fungi (–AM), colonized by arbuscular mycorrhizal fungi (+AM); Ca (calcium); POD (peroxidase); CAT (catalase); SOD (superoxide dismutase); CO_2_ (carbon dioxide); K (potassium); N (nitrogen); P (phosphorus); ROS (reactive oxygen species); EPS (extracellular polymers).

Plants and their associated rhizosphere microbes engage in a bidirectional feedback loop mediated by root exudates, particularly flavonoids. Under drought stress, tolerant plants can recruit beneficial microbial communities (e.g., specific rhizosphere fungi) by releasing signaling molecules such as sugars, amino acids, and flavonoids [314, 315]. In return, these recruited microbes provide feedback to the plant in the form of plant hormones, volatile organic compounds (VOCs) [316], and quorum‐sensing molecules, which regulate plant hormone pathways and promote flavonoid synthesis [317], thereby enhancing drought resistance and influencing carbon and nitrogen cycles [318, 319, 320] (Figure 5). However, drought can also disrupt this root microbiome communication by altering the quantity and composition of root flavonoid exudates [321] (Figure 5), which in turn reshapes the plant microbiome through metabolic responses and ecological feedback [319]. Understanding this flavonoid‐mediated feedback loop opens opportunities for bioremediation‐like strategies to enhance drought tolerance [254, 255, 322, 323, 324].

### 6.1 The Significance of Plant‐Microbe Interactions in Drought Conditions

Improving soil microbial community characteristics and microbial activity in arid areas could be used in improving drought resistance for example via shaping the soil characteristics or by inoculation of beneficial microbes (Table 3). For example, biological soil crusts, surface soil communities composed of cyanobacteria, lichens, mosses, and fungi, have unique capacities for nitrogen fixation, soil aggregation, and degraded soil rehabilitation, and integrating phylogenetic diversity with functional trait analysis can identify keystone microbial taxa [325, 326]. Inoculation of beneficial bacteria can also alleviate plant performance during drought stress, as shown with sorghum (*Sorghum bicolor* L.) plant and *Monoderm sp*. Bacteria [196]. Moreover, developing a biological inoculant combining various microbial genera or strains would likely provide efficient tool in improving drought tolerance, as the microbes may have complimentary effects or such mixture can enhance the likelihood of successful survival and colonization of the introduced microbes. For example, co‐inoculation of multiple strains of bacteria and fungi has been proven to significantly improve the soil water retention and plant physiological traits, revealing a synergistic “soil‐root‐microbe” network that can enhance the drought resistance of agricultural systems [327]. This highlights the potential of rhizobacterial inoculations as a tool to reduce drought‐induced oxidative stress. Nanoparticle‐microbe seed coating could also be used as a tool to improve the establishment of microbial inoculums, as this method has been shown to protect microorganisms and metabolites, maximizing their potential [328].

**TABLE 3:** Representative soil microorganisms and their agricultural application.

Microbial species	Representative bacteria	Major crop hosts	Function	Related research
AMF	*Glomus* spp.	Wheat, rice, maize, soybean, tomato	Enhance P uptake, improve water use efficiency, drought tolerance	[44, 45, 249, 250]
PGPR	*Pseudomonas fluorescens*	Wheat, sorghum	Promote N, P, K uptake; produce phytohormones; biocontrol	[175, 329]
Actinobacteria	*Streptomyces* spp.	Straw	Decompose organic matter; produce antibiotics against pathogens; promote growth	[330]
Bacteria	*Bacillus* spp.	Wheat, maize, tomato, sorghum	N fixation, P solubilization, produce stress‐protective substances (e.g., proline)	[375]
Fungi	*Saccharomyces cerevisiae*	Wheat, rice, maize, tomato, soybean, chickpea	Promote organic matter decomposition, improve soil structure, enhance nutrient availability	[331]
Algae	Cyanobacteria	Rice, wheat	Biological N fixation, improve soil structure, form biological soil crust	[214]

*Note*: ND, no data

Moreover, by engineering root exudates it could be possible to selectively enrich probiotics with quorum sensing (QS) activity and together with the development of QS signal molecule encapsulation technology it could be possible to aid plant's ability to recruit stress‐resistant microbial communities [332]. QS refers to the way in which microorganisms generate, release, and sense specific chemical signaling molecules for cell‐to‐cell chemical communication. QS forms a synergistic network with mycorrhizal symbiosis and flavonoid metabolism through signal crosstalk, functional complementarity, and technical integration, acting together at the “plant‐microbe‐soil” interface. Bacterial generas *Streptomyces* and *Bacillus* can improve the colonization and effectiveness of probiotics in plants [333], which under drought stress enhances plant water status via induced systemic tolerance. *Streptomyces* forms hypha‐like network around roots that extends the root's absorptive surface area, allowing access to deeper soil water [334]. Although *Streptomyces* lacks motility, it can be transported into plant tissues via flagellar interactions with other bacteria [335], facilitating the delivery of drought‐protective functions such as siderophore production and phytohormone synthesis to alleviate water deficit.

Besides Streptomyces and Bacillus, PGPR include a wide range of genera, including for example Azotobacter, Enterobacter, Achromobacter, Bradyrhizobium, Serratia, Agrobacterium, Phyllobacterium, Bacillus, Acinetobacter, Arthrobacter, Pseudomonas, Azospirillum and Rhizobium [336], and are promising candidates as microbial inoculants to combat drought stress. PGPR enhance plant drought tolerance by improving the uptake of nitrogen, P, and k, as well as by regulating phytohormone levels (e.g., gibberellins (GA), ABA), reducing ethylene concentrations, modifying the expression of drought‐resistance‐related genes (e.g., DREB, LEA, and aquaporins) and root architecture, and by activating the synthesis of antioxidant enzymes [337]. For example, Research on sorghum (Sorghum bicolor L.) inoculated with PGPR reveals improved drought resilience, attributed to elevated antioxidant activity, increased GA, IAA, and cytokinin levels, adaptive root morphological changes, and the activation of brassinosteroid, SA, (JA, and sphingolipid signaling pathways [329]. Currently, Rhizobium/Bradyrhizobium and Azospirillum inoculants are being used in agriculture in arid areas to promote nitrogen supply and early drought resistance (e.g., commercial soybean (Glycine max) rhizobia formulations in semi‐arid Mexico [338]). Beneficial microbes such as Pseudomonas sp. and Bacillus subtilis secrete extracellular polymeric substances (EPS) that bind soil particles into water‐stable microaggregates, increasing soil structural stability [339], and therefore are good candidates as microbial inoculums in arid areas.

Besides PGPBs, inoculation or improvement of colonization of AMF provide a promising tool in improvement of drought tolerance as AMF stimulate lateral‐root proliferation and increase root‐hair density of the host plant, strengthening root–soil anchorage and improving soil resilience to erosion [340, 341], they could be used to wider extent in bioremediation of arid areas. In fact, some AMF‐based products are widely used in rice (*Oryza sativa* L.) production in India to stem the effects of low P levels in the soil and rising costs of synthetic phosphate fertilizers [331]. Therefore, we propose that deploying PGPR–AMF consortia that combine nitrogen‐fixing bacteria (e.g., *Rhizobium*, *Azospirillum*) with AMF offers a promising strategy to enhance drought resistance in aridregion crops by improving root water uptake, stabilizing soil structure via EPS secretion, and facilitating early plant establishment.

Increase in proline levels via suitable microbial inoculants may also improve drought tolerance, because proline is one of the most important osmolytes accumulated by plants under drought stress. PGPR enhance proline accumulation through upregulation of biosynthesis genes (*P5CS*, *P5CR*) and suppression of catabolism (*ProDH*) [342]. A study on cucumber (*Cucumis sativus* L.) used three PGPR strains [343]: *Bacillus cereus* AR156, *Bacillus subtilis* SM21, and *Serratia sp*. XY21. The treatment increased leaf proline content three to fourfold relative to untreated controls, enabling effective osmotic adjustment under drought stress.

In summary, microbial agents can enhance plant drought resistance or alleviate drought stress through various mechanisms [344], including the formation of biofilms, release of EPS, regulation of plant hormones, and antioxidant systems. Moreover, biofilms could also be used in improving drought resistance due to their ability to bind soil particles through the hydrophilic properties of EPS, reducing pores and forming a water‐retaining barrier, while promoting the formation of agglomerates inhibiting water evaporation [345, 346]. Moreover, cyanobacterial‐dominated biological soil crusts could also be utilized, as these surface soil layers regulate water infiltration and retention, thereby optimizing soil hydrological processes [347]. Therefore, combining microbial inoculants would be an important tool to improve erosion resistance in arid regions.

### 6.2 Flavonoids and Their Relationship With Microorganisms

Flavonoids can modulate the colonization process of mycorrhizal fungi, affecting hyphal growth and branching, and their antioxidant properties can alleviate oxidative stress generated during mycorrhizal symbiosis, protecting plant and fungal cells from damage [311], which has important application value in agriculture and ecosystems. Therefore, addition of flavonoids could be used as a bioremediation method to improve mycorrhizal symbiosis and thus aid drought resilience. For instance, addition of micromolar levels of flavonoids (chrysin, quercetin, rutin) to the roots of tomato (*Solanum lycopersicum* L.) plants has been shown to significantly enhance spore germination and internal colonization of the AMF *Rhizophagus irregularis*, with colonization rates two to four times higher than controls, while also reducing ROS generated during AMF colonization, improving antioxidant enzyme activity, flavonoid content and water use efficiency [348]. This finding not only confirms the dual role of flavonoids in the establishment of mycorrhizal symbiosis, as both signaling molecules and protective agents, but also provides direct evidence for utilizing flavonoid‐AMF composite formulations to enhance crop resilience in sustainable agriculture [348]. Similar strategies have been successfully applied in other crop systems: in maize (*Zea mays* L.), exogenous application of rutin significantly reduced oxidative damage and enhanced osmolyte accumulation under osmotic stress [150]. In potato (*Solanum tuberosum* L.), formononetin seed treatment increased AMF spore formation by threefold and reduced P fertilizer requirements by 50% [349]; and in wheat (*Triticum aestivum* L), foliar application of vitexin activated phenylpropanoid pathway genes and improved drought tolerance [350]. These cases demonstrate that flavonoids can be delivered through multiple approaches—including root application, foliar spraying, and seed coating—to enhance crop‐AMF symbiosis across diverse agricultural systems. The antioxidant properties of flavonoids can scavenge ROS and protect cell membrane integrity and may indirectly enhance aquaporin function by maintaining membrane lipid stability. AMF symbiotic signals (such as strigolactones) can directly activate the expression of root aquaporin genes to improve water uptake efficiency [351], and act synergistically with the accumulation of osmotic adjustment substances including proline and soluble sugars to maintain cell turgor under drought stress [352]. Meanwhile, AMF can promote the biosynthesis and accumulation of flavonoids, which together with antioxidant enzymes such as SOD and CAT form a dual defense barrier to effectively scavenge drought‐induced reactive oxygen species and alleviate oxidative damage [313, 352].

Numerous crop trials as summarized above verify that exogenous flavonoids exert positive regulatory effects on arbuscular mycorrhizal symbiosis and collaboratively strengthen crop drought tolerance. Nevertheless, the regulatory modes of rhizosphere microbial recruitment mediated by flavonoids are inconsistent across crop varieties, exhibiting coexisting interspecies conserved traits and genotype‐specific divergence.

The core signaling cascade whereby root‐exuded flavonoids drive chemotaxis and root colonization of beneficial rhizosphere microbes is evolutionarily conserved in major cultivated crops. Under drought stress, however, interspecific and intraspecific genotypic disparities alter flavonoid secretion profiles and reshape the assembled rhizosphere microbiome [353]. Root‐released flavonoids function as chemical messengers to facilitate symbiotic associations between crops, arbuscular mycorrhizal fungi and plant growth‐promoting rhizobacteria, representing a universal drought‐defensive strategy for cultivated plants. Even so, distinct crop lineages synthesize specialized flavonoid metabolites to match their dominant symbiotic partners: leguminous crops produce isoflavones to initiate *rhizobial* nodulation; cereal staples secrete flavones such as apigenin and tricin to enrich *Oxalobacteraceae* and Pseudomonas; solanaceous and cucurbitaceous vegetables primarily release quercetin‐type flavonols to accelerate AMF hyphal extension and root colonization [354]. Within a single crop species, genetic variations modify the yield and compositional proportion of root flavonoid exudates, which impose divergent selective pressures on rhizosphere microbial communities. For instance, nitrogen‐efficient rapeseed cultivars secrete substantially higher amounts of kaempferol than low‐efficiency counterparts, selectively enriching *Bacillaceae* populations and enhancing crop stress resilience [170]. Such genotype‐specific metabolic discrepancies arise from variable transcriptional intensities of *MYB* transcription factors and pivotal phenylpropanoid biosynthetic genes across germplasm resources. Unique host‐specific rhizosphere microbial assemblies can therefore form even under identical water‐limited cultivation conditions [355]. Collectively, the fundamental signaling framework supporting flavonoid‐guided microbial recruitment is conserved across crop taxa, yet fine‐scale secondary metabolite signals and resultant microbial community structures are strongly genotype‐dependent. This genetic variation creates uneven drought adaptability of plant‐microbe holobionts among different cultivars and crop species, which further restricts the broad popularization of flavonoid‐targeted microbial regulation techniques in agricultural production [356].

## 7 Future Research Direction

### 7.1 Study on Genetic Mechanism of Drought Tolerance

With the progress of genotyping technology, genome wide association studies (GWAS) can reveal the molecular genetic mechanism behind natural phenotypic variation [357].This technology could be used to broader extent to detect drought sensitive and drought resistant genotypes, for example by comprehensively identify gene families and their expression patterns related to flavonoid or other secondary metabolites synthesis under drought stress, helping to elucidate the drought‐resistant mechanisms in plants. By constructing a gene regulation network, the mechanisms of the coordinated regulation of key transcription factors in the flavonoid biosynthesis pathway can be determined [358, 359]. This information could be used to improve plant drought tolerance [360]. For example, GWAS analysis has pinpointed TaPP2C6 as a major negative regulator of wheat (*Triticum aestivum* L.) drought tolerance [361]. Knockout of this gene improves drought resistance without compromising yield, and a superior allelic variant has been characterized. Furthermore, integrating machine learning with GWAS could help establish predictive models for yield and drought resistance, as demonstrated in the same study [361], providing important support for breeding high‐yield and drought‐tolerant varieties. As the genomes of various crops are relatively well studied and are available in public repositories, these databases could be to wider extent to find candite genes using statistical or bioinformatic approaches. A meta‐analysis integrating 511 quantitative trait loci (QTL) data from 27 independent studies identified 83 drought‐associated meta‐QTL (MQTL), including 20 core MQTL in maize (*Zea mays* L.) [362]. Further analysis uncovered 63 maize‐rice orthologous genes and 583 candidate genes, highlighting the central function of phytohormone signaling pathways in drought responses. These results deliver precise targets and theoretical guidance for genetic improvement of maize (*Zea mays* L.) drought tolerance.

In addition to triggering instantaneous transcriptional reprogramming under drought conditions, water deficit reshapes plant epigenetic landscapes, thereby conferring long‐term and heritable effects on drought responses in subsequent generations. Deciphering epigenetic signatures represented by dynamic DNA methylation patterns offers innovative breeding approaches that complement or even substitute conventional drought improvement strategies [363]. Methylation signatures established upon initial drought exposure strengthen plant environmental fitness and establish stable stress “memory,” which continuously modulates the transcription of stress‐responsive genes when plants encounter recurring drought episodes [364]. Such epigenetic remodeling is synergistically governed by drought‐triggered regulatory cascades. ABA‐activated transcription factors *ABF3/4* and *GIGANTEA* not only accelerate floral transition through upregulating *CO1* and *FT* expression [116] but also boost flavonoid biosynthesis to shield reproductive tissues from drought damage. Likewise, nuclear factor Y (NF‐Y) transcription factors reinforce drought tolerance by synchronizing proline accumulation and terpenoid metabolism, redirecting carbon allocation from vegetative growth to stress defense pathways [365]. Rice (*Oryza sativa* L.) based research has validated that drought‐induced DNA methylation modifications can be stably transmitted for a minimum of two generations via the RNA‐directed DNA methylation (RdDM) pathway [366]. Accordingly, progeny subjected to repeated drought exposure can rapidly mobilize ABA signal transduction, flavonoid production and antioxidant defense systems, maintaining superior photosynthetic capacity and stable grain yield.

To cope with drought stress, plants activate a full spectrum of epigenetic regulatory machinery encompassing DNA covalent modification, histone modification, histone variants, ATP‐dependent chromatin remodeling, histone chaperones, small RNAs, long non‐coding RNAs and other uncharacterized pathways, all of which jointly adjust chromatin accessibility and transcriptional activity of flavonoid biosynthetic genes [367]. Based on this understanding, a recent study proposed a CRISPR‐dCas9‐H3K27me3 targeted writing approach, in which sgRNAs guide a dCas9–CLF/SWN complex to drought‐negative regulatory genes (e.g., *CYP707A*, *PIP2;1*) [368]. A transient H3K27me3 repressive mark is deposited during a mild‐drought or ABA pulse applied at the seedling stage, establishing a transgenerational drought epigenetic memory without altering the DNA sequence and thereby enhancing crop drought tolerance. In plants, DNA methylation occurs at CG, CHG, and CHH sequence contexts [369]. In *Arabidopsis*, methylation is maintained by three methyltransferase genes: *MET1* (CG), *CMT3* (CHG), and *DRM2/CMT2* (CHH) [370, 371]. From a breeding perspective, natural variation in these genes can be exploited through marker‐assisted selection, or they can be directly edited via CRISPR to modulate methylation patterns and enhance drought tolerance. Beyond DNA methylation, Polycomb repressive complexes (PRCs) offer additional targets for epigenetic modifications. *LHP1*, a component of PRC‐like complexes, balances plant development and stress responses and can be edited via CRISPR‐Cas9 to improve drought tolerance [372, 373]. PRC2, which deposits H3K27me3 repressive marks, regulates ABA signaling and flavonoid synthesis. Mutants affecting PRC2 function show improved drought survival, suggesting that manipulating H3K27me3 deposition, for example, through CRISPR‐dCas9‐based targeted writing, could also enhance stress resilience [374, 375].

As drought stress enhances the accumulation of certain plant metabolites, these could be used as biomarkers for detecting genotypes with better drought tolerance for breeding purposes. In soybean (*Glycine max*), drought stress modifies isoflavone distribution, suggesting that isoflavonoid metabolism is closely associated with drought adaptation and can serve as a metabolic biomarker for breeding drought‐resistant cultivars [376]. Similar examples exist in other crops. In barley (*Hordeum vulgare* L.), drought‐induced accumulation of flavonoids such as luteolin and apigenin derivatives has been correlated with oxidative stress responses and can serve as selection markers for drought‐tolerant genotypes [38]. In wheat (*Triticum aestivum* L), drought stress induces elevated levels of phenolic acids (e.g., ferulic acid) as well as other metabolites such as proline and tryptophan. These droughtresponsive compounds have been proposed as biomarkers for screening tolerant genotypes [377]. Similarly, in rice (*Oryza sativa* L.), metabolites such as proline and trehalose have been used as indicators of drought tolerance in breeding programs. Trehalose metabolism, in particular, serves as a biochemical marker that distinguishes tolerant from sensitive varieties [378]. Advanced structural biology tools, such as X‐ray crystallography and cryo‐electron microscopy, can further aid breeding efforts by enabling systematic characterization of flavonoid biosynthetic enzymes (e.g., *PAL*, *CHS*, *FLS*) and identification of their active sites and catalytic mechanisms [379]. Post‐translational modifications also play a role in drought‐regulated flavonoid synthesis, affecting enzyme activity, protein stability, and intermolecular interactions [380, 381]. Combining enzyme engineering, transgenic technology, and conventional breeding could facilitate the selection of crop varieties with high flavonoid content and improved drought tolerance [382]. However, knowledge gaps remain in understanding how plants synthesize flavonoids under different environmental conditions, limiting the application of this knowledge to improve crop stress resistance. Further research is needed to fill these gaps.

### 7.2 Application of Nanomaterials

**FIGURE 6:**
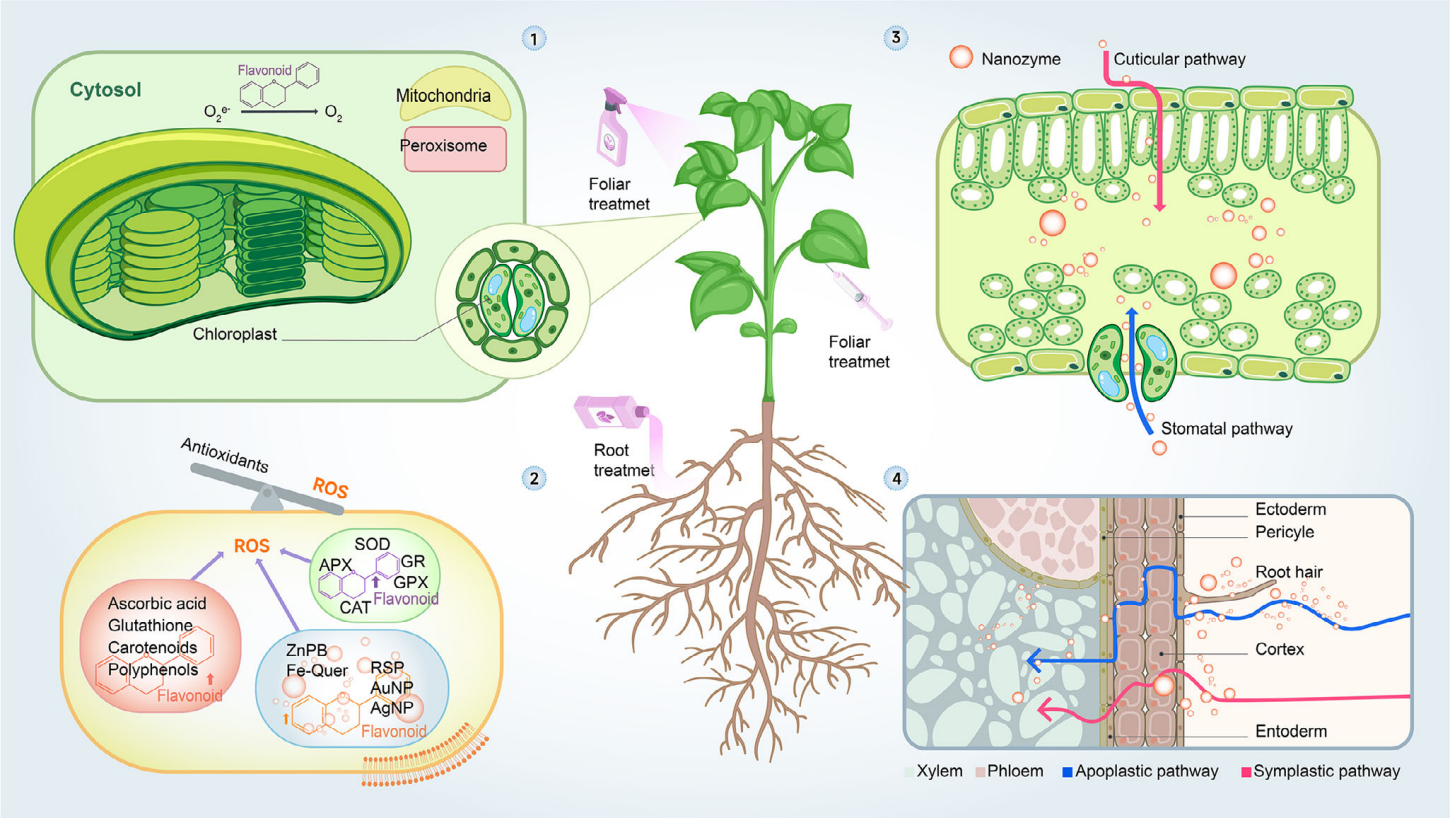
Foliar and root application of nanoparticles and their involvement in drought tolerance. ① When plants are subjected to environmental stresses such as drought, high temperature, and strong light, photosynthesis is affected and ROS increase. During this time, the antioxidant system in the cells can scavenge ROS and maintain the normal physiological functions of the cells. ② ROS scavenging systems in the cytosol and peroxisomes. Once inside plant cells, nanoparticles remove ROS through three complementary mechanisms: Natural antioxidant enzymes (green). These include superoxide dismutase (SOD, converts O_2_^−^ to H_2_O_2_), ascorbate peroxidase (APX), catalase (CAT), glutathione peroxidase (GPX), and glutathione reductase (GR). These enzymes convert ROS into water and oxygen, protecting cells from oxidative damage under drought stress; Non‐enzymatic antioxidants (orange). These include ascorbic acid, glutathione, carotenoids, and polyphenols. They directly neutralize ROS and help maintain cellular redox balance, reducing oxidative damage to chloroplasts and mitochondria; Synthetic nanozymes (blue). These are engineered nanomaterials with enzyme‐like activity, including ZnPB (zinc‐based Prussian blue analogue), Fe‐Quer (iron‐quercetin complex), RSP (redox‐sensitive polymer), AuNP (gold nanoparticles), and AgNP (silver nanoparticles). These nanozymes mimic natural antioxidant enzymes to scavenge ROS and improve drought tolerance. ③ After entering through the cuticle and stomata of plant leaves, nanoparticles can participate in the transport of nutrients or signaling substances; ④ Nanoparticles enter the plant roots through the symplastic pathway and apoplastic pathway for nutrient transport.

Under drought stress, both leaves and roots of plants suffer from oxidative damage. Nanomaterials offer a strategy to alleviate this stress. Nanoparticles can enter leaves through stomatal pores to mitigate drought stress in above‐ground tissues [383], with entry efficiency depending on particle size [384]. Once inside, nanozymes, nanomaterials with enzyme‐like catalytic activity, scavenge ROS, protect chloroplasts, and improve photosynthesis [385, 386, 387] (Figure 6). Nanozymes can also mimic natural enzymes involved in secondary metabolism, catalyzing the oxidation or polymerization of flavonoid precursors (e.g., phenylpropanoids), thereby promoting flavonoid synthesis [388]. Encapsulated nanozymes have been developed for nutrient delivery, further enhancing plant stress tolerance [389], making them promising candidates for developing drought‐tolerant crop varieties [390, 391].

In root application or seed treatment, nanoparticles enter roots via apoplastic or symplastic pathways [392] (Figure 6). Once inside the root system, they can be transported to shoots or act locally to reduce ROS, improve root architecture, enhance water and nutrient uptake, and further promote flavonoid synthesis to activate antioxidant defenses, helping plants maintain root function under water deficit [393, 394].

For example, iron–quercetin nanozymes (quercetin conjugated with iron ions) mimic the activities of SOD, CAT, and POD to scavenge ROS [395]. Luteolin encapsulated in exosome‐like nanovesicles (Exo@Lu) improves luteolin solubility and stability, thereby enhancing drought tolerance [396]. The nano enzyme prepared by leaf extract combined with AuNP and AgNP provides us with a more environmentally friendly method for synthesizing nano particles [397]. Conjugating flavonoids with nanozymes generates green nanocatalysts that scavenge ROS and stimulate further flavonoid release, boosting stress resilience [398]. In wheat (*Triticum aestivum* L), co‐application of PGPR and zinc oxide nanoparticles (ZnO NPs) enhanced drought tolerance by increasing indole acetic acid, protein, and soluble sugar contents [399]; seed dressing with ZnPB nanozymes increased wheat yield by 12.15% under drought stress [400]. In tomato (*Solanum lycopersicum* L.), a ROS‐responsive star polymer (RSP) reduced ROS accumulation, enhanced photosynthesis, and provided carbon skeletons for flavonoid synthesis [389].

Although plant‐derived extracts offer a more environmentally friendly route for nanoparticle synthesis [401], the widespread adoption of flavonoid–nanoparticle technologies faces several challenges. First, most studies remain at the laboratory or greenhouse scale, with limited field validation. Second, the long‐term environmental fate and toxicity of nanoparticles in soil and plant tissues are not fully understood. Third, the production technology and cost of nanoencapsulation and functionalized nanozymes limit commercial scalability. Therefore, further research is needed to advance field applications.

## 8 Conclusion

This review provides an in‐depth exploration of how globally important economic and food crops such as maize (*Zea mays* L.), wheat (*Triticum aestivum* L), and soybean (*Glycine max*) maintain physiological function, yield stability, and nutritional quality via secondary metabolites under drought stress. Such information is critical in batting a major global environmental concern exacerbated by climate change. Flavonoids play a key role in regulating plant growth and in defending against biotic and abiotic stresses. They enhance drought tolerance by modulating ROS levels and stomatal movement through the up‐regulation of flavonoid‐biosynthesis genes. Therefore, increasing endogenous flavonoid content in plants through breeding and genome editing, or regulating beneficial microbes via exogenous approaches such as soil amendment and nanocarrier delivery, provides feasible strategies to enhance drought resistance, ensure yield, and maintain nutritional quality in cereal crops.

Soil improvement is crucial for enhancing plants' drought resistance. Green organic amendments improve soil water retention and promote beneficial microbial activity, creating a more favorable growth environment for plants. Advanced irrigation techniques, such as magnetized and nanobubble irrigation, not only optimize soil moisture conditions but also increase the flavonoid content in plants, thereby enhancing their tolerance. Plants that have endured severe drought develop “drought memory,” which can affect their offspring. This together with better understanding of epigenetic mechanisms, needs further attention in aiding methods in developing more drought resistant varieties.

New metagenomics and high‐resolution measurement techniques can deepen our understanding of drought‐responsive metabolites, providing a powerful tool for the analysis and identification of flavonoids and other secondary metabolites. These methods can be applied more widely to explore the “stress memory” of plants and physiological responses under drought conditions to better understand the genetic, metabolomics, epigenetics and other molecular mechanisms of plant drought resistance, thereby facilitating germplasm screening and selection of suitable cultivar in arid areas. Nanomaterials also play a role in delivering active ingredients flavonoids, (e.g., luteolin, quercetin) within plant cells, reducing oxidative stress, and promoting cellular metabolism and repair due to their unique properties. Integrating these strategies enables the establishment of an efficient technical framework based on plant physiological and molecular responses to drought stress, offering a systematic approach to improve drought resistance, yield stability, and nutritional quality of major food and economic crops, with important scientific significance and application prospects for addressing global climate change, ensuring food security, and promoting sustainable agricultural development.

## 9 Future Perspective

Climate change‐induced abiotic stresses, particularly drought, poses an urgent threat to global food security and agricultural sustainability, highlighting the need for translational research that integrates plant metabolism, microbial ecology, and agricultural technology. Future research should prioritize deciphering the species‐specific functions of flavonoids and other PSMs, and elucidate the mechanisms underlying the maintenance of crop yield stability and nutritional quality under drought stress. This requires the integration of multi‐omics approaches including genomics, metabolomics, and microbiomics to unravel context‐dependent interactions between plant secondary metabolites and rhizosphere microbes (e.g., AMF, plant growth‐promoting rhizobacteria, actinobacteria), with a focus on the coordinated optimization of nutrient acquisition and drought resistance under field conditions.

As the foundation of sustainable crop production, soil health urgently demands the development of high‐performance green organic amendments and precision irrigation technologies suitable for arid and semi‐arid agricultural systems (e.g., magnetized irrigation, nanobubble irrigation). These innovations can not only improve soil water‐holding capacity, aeration, and fertility, but also synergize with plant–microbe interactions to promote root development and secondary metabolite biosynthesis, ultimately enhancing crop adaptability to water deficit.

In the field of nanobiotechnology, nanozymes and nanocarriers exhibit great potential in the targeted regulation of plant redox homeostasis and flavonoid metabolism. Future research must address key bottlenecks for field application: optimizing the catalytic specificity of nanozymes through biomimetic design (e.g., developing non‐metallic materials to avoid non‐specific reactions), improving their biocompatibility in crop cells, and exploring synergistic effects between nanomaterials and endogenous/exogenous plant secondary metabolites to construct efficient and green nano‐bio hybrid catalytic systems.

Overall, the interdisciplinary integration of molecular biology, microbial ecology, nanotechnology, and agronomy is essential to translate basic theories into practical application strategies. Future research should prioritize the development of field‐validated and scalable solutions to enhance the drought resistance of staple crops, safeguard global food security, and promote sustainable agricultural development under climate change.

## Author Contributions

**Xiaoyi Duan**: writing – original draft, methodology, validation, writing – review and editing, software, visualization, formal analysis, data curation. **Litao Wang**: funding acquisition. **Tuuli‐Marjaana Koski**: supervision, writing – review and editing. **Lizhe An**: resources, conceptualization. **Thomas Efferth**: resources, conceptualization. **Yujie Fu**: resources, project administration, funding acquisition, supervision, conceptualization.

## Conflicts of Interest

The authors declare no conflicts of interest.

## Data Availability

The authors have nothing to report.
